# Availability and spatial distribution of crop and forest biomass residues for biochar production in Kenya

**DOI:** 10.1038/s41598-026-42350-0

**Published:** 2026-03-02

**Authors:** Timothy Namaswa, David F. R. P. Burslem, Jo Smith, Waheed Afzal, Jennifer Wardle, Nellie Oduor, Leonard Kubok, George Muthike, Faith Malei

**Affiliations:** 1 School of Biological Sciences, University of Aberdeen, St Machar Drive, Aberdeen, AB24 3UU Scotland, UK; 2 Kenya Forestry Research Institute, P. O. Box 64636–00620, Nairobi, Kenya; 3 Interdisciplinary Institute, University of Aberdeen, King’s College, Aberdeen, AB24 3FX Scotland, UK; 4 School of Engineering, University of Aberdeen, Fraser Noble Building, Aberdeen, AB24 3UE Scotland, UK; 5 Ministry of Agriculture and Livestock Development KE, P. O. Box 30028-00100, Nairobi, Kenya

**Keywords:** Biochar, Crop residues, Forest residues, Spatial distribution, Economic residues, Ecology, Ecology, Environmental sciences, Plant sciences

## Abstract

Uptake of biochar for fuel briquetting and soil amendment is constrained in sub-Saharan Africa by inadequate knowledge of the quantity and distribution of feedstocks. This study assessed the quantities and spatial distribution of crop and forest residues available for biochar production in Kenya based on productivity data from 2021 and 2022. The residues were quantified using residue product ratios, surplus available factors and economically viable factors. Kenya produces (0.5–2.4) × 10^7^ Mg y^−1^ of crop residues and (1.48–1.8) × 10^5^ Mg y^−1^ of forest residues that are potentially available for biochar production. While crop and forest production are the core drivers of the availability of economically viable residues, residue to product ratios and surplus available factors are the primary drivers of residue densities. Crop residues were concentrated in counties located in western, central and southern Kenya. While all counties possess diverse types of residues, maize stalks were prevalent in all 47 counties. No county satisfied the combined requirements of high amounts of residues, high residue density and low supply uncertainties. Therefore, although Kenya has abundant and diverse residues that could produce economically viable biochar, locating production facilities will require a trade-off between counties with high residue densities, or those with supply uncertainty.

**Supplementary Information:**

The online version contains supplementary material available at 10.1038/s41598-026-42350-0.

## Introduction

Biochar production and utilization has become an important subject of research because of its useful properties, broad applications and promising development prospects^1^. This is evidenced by the cumulative number of scientific publications, indexed in “Web of Science”, increasing from < 50 in the early 2000s to > 3500 by 2020^2^, with a search by the current team producing > 46,000 by 2025. These publications demonstrate the diverse opportunities to apply biochar to provide sustainable solutions to waste management and mitigate climate change impacts^1^. This increasing awareness is, in turn, creating demand for biochar and biochar products. The global biochar market is projected to grow at a compound annual rate of about 17.0% by revenue and 11.0% by mass between 2019 and 2028^3,4^.

Africa holds potential for biochar market growth because of factors including rapid population growth, increasing food demand and over-dependence on biomass energy from declining forest resources. Further drivers are increasing levels of soil degradation, which create a vicious cycle of poverty among smallholder farmers, the widespread availability of diverse biomass wastes for biochar production and the need for a circular economy to reduce imports and enhance self-sufficiency^5^. Despite all of these factors favouring market growth for biochar, the African market is only expected to grow at a rate equivalent to the global rate, a compound annual growth rate of 16.8% by revenue and 11.7% by mass during the fiscal years 2019 to 2028^6^. This slow growth can be attributed to the limited development of biochar production facilities in Africa, due to lack of evidence from research on optimal locations for processing facilities, and inadequate legislative and institutional frameworks that could help to lobby for and support sustainable and profitable biochar production^5^.

In Kenya, production of biochar is sustainable and feasible due to the potential biomass residues from forests and agricultural activities^7^. Crop production in Kenya is expected to increase by 40% by 2030 based on a 2015 baseline of 26.7 × 10^6^ Mg^8^. The increasing crop production could be due to improving market access and better prices that encourage more people into farming, and increasing investments in climate smart agricultural systems, such as improved drought resistant crop varieties, adoption of alley cropping and up-scaling of small-scale irrigation^9^. Although Kenya is yet to establish targets for wood production from industrial and on-farm plantations, Ototo and Vlosky^10^ identify that the country produces a substantial amount of wood. Globally, wood production from industrial and on-farm plantations is expected to increase by about 45–65% of values in 2000 by 2050^11^. These global predictions fit well for Kenya, as the government’s target is to increase national tree cover to at least 30%, by 2032, from the current tree cover of 12.4%^12^. An increase in crop and wood production will result in increased amounts of biomass residues, which create opportunities for value addition including biochar production.

Sustainable production of high-quality biochar requires stable and profitable biochar production plants to be established. Such plants should be close to sources of adequate raw material and have easy access to markets^13^. These criteria are important as collection and transportation costs affect the economic feasibility of residue management through utilization^14^. In Belgium, for example, it costs approximately €7.20 to transport 1 Mg of tree bark residues with a value of €4.00 over 100 km^15^. Kimutai et al.^16^, Suryani et al.^17^, Wekesa^18^ and Welfle et al.^19^ determined the potential of crop residues for bioenergy production and mapped areas where major crops, such as maize, cassava, barley and rice are grown in Kenya. However, they assumed that all crop residues in Kenya can be used for bioenergy production, therefore failing to consider competing uses of these resources, such as consumption by livestock and applications in soil improvement and industry, leading to over-estimation of the potential of crop residues for bioenergy. These estimates, therefore, ignore the perspective that modern bioenergy production should focus on surplus residues to minimize negative impacts on the environment and farming households^20,21^. In addition, previous studies have not included forest residues in their estimation and mapping, except for Suryani et al.^16^, who estimated the potential of sawdust only. The absence of forest residue data presents a significant knowledge gap with serious implications for policy-making, and effective planning for residue utilization. A significant amount of roundwood used in Kenyan sawmills ends up as residues due to low utilization efficiency (28%)^10^, and this could represent an important source of biomass residues for biochar production. Therefore, Kenya lacks a comprehensive and reliable assessment of available biomass residues, their geographical distribution and current usage patterns. This knowledge gap is a major technical hindrance to the development of residue processing in many sub-Saharan African (SSA) countries^22,23^. Consequently, there is need to develop and apply a method that combines both crop and forest residues for better residue management. In this paper we present this approach to estimating and mapping the distribution of biomass residues in Kenya to serve as a case study for broader application across Sub-Saharan Africa.

Resource-based quantification, which uses the total amount of product harvested and the ratio of residue to total production, is the main approach used to assess availability of resources such as biomass residues^24^. Geographical information systems (GIS) can be used to visualize the spatial distribution of estimated biomass residues^14,19^. A combination of resource-based and GIS approaches has been used to estimate biomass residues and their spatial distribution in different parts of the world, including SSA countries such as Zambia and Nigeria^14,19^. In Kenya, Wekesa^18^ determined the potential energy from major crop residues in Western and Rift-valley regions and mapped sites for biomass power plants using GIS. Therefore, the purpose of this study was to provide a comprehensive assessment of available crop and forest residues and their spatial distribution across the whole of Kenya to foster development of viable residue processing plants. We tested the hypothesis that available crop and forest residues are characterized by spatial heterogeneity at county level, reflecting consistent bioclimatic variation through time that enables recommendations for the optimal location of residue processing facilities. Specifically, the objectives were to a) determine the available amount of crop and forest residues that could produce economically viable biochar in Kenya, b) map the spatial distribution of economically feasible residues from crops, and c) identify the main types of residues that could drive biochar production in each of the 47 counties in Kenya. The results are expected to guide policy and decision-making towards enactment of supportive legislation and creation of institutional frameworks, that will support sustainable and profitable biochar production for sustainable development and livelihood improvement in Kenya.

## Materials and methods

### Study site and data collection

The study was conducted in Kenya (5° 40’ N—4° 40’ S, 33° 50’—41°45’ E). The country has both a national government and 47 semi-autonomous county governments. Kenya experiences a varied mean annual temperature ranging from 12ºC in the higher elevations to over 30ºC in the coastal and northern parts, with mean annual rainfall ranging from 600 mm to over 2000 mm^25^. There is also a significant variation in soil types, topography and vegetation depending on altitude and degree of aridity^25^. Kenya is a lower-middle income country with an economy that is heavily dependent on agriculture, contributing over 26% of annual gross domestic product directly, and another 27% indirectly through linkages with other sectors^26^. This study estimated crop and forest residues from production data collected over a period of two calendar years (2021 and 2022), which were selected because they were the most current at the start of our data collection in 2023. Annual crop production data in each of the 47 counties in Kenya were obtained from the Ministry of Agriculture and Livestock Development (MoALD), and 2023 and 2024 Agriculture and Food Authority (AFA) reports. National forest production data were obtained from Food and Agriculture Organization statistics.

### The potential of crop residues in Kenya

#### Gross crop residue potential in Kenya

Gross available residues refer to the total amount of residue that can be obtained from harvested yields at a specified location, where production depends on farming practices and environmental factors such as soil properties and climate^14,27^. The gross residue production across every type of crop residue was estimated using Eq. 1.

1 $$R_{ciky} = \mathop \sum \limits_{i = 0}^{n} P_{ckjy} \times w_{ci} \times \mu_{cik}$$

Where for the ith type of residue from kth crop for the ${\mathrm{yth}}$ year, ${\mathrm{R}}_{{{\mathrm{ciky}}}}$ is the gross residue production (Mg), ${\mathrm{P}}_{{{\mathrm{ckjy}}}}$ is the crop production (Mg), ${\mathrm{w}}_{{{\mathrm{ci}}}}$ is the moisture content of the residue at the time of production, ${\upmu }_{{{\mathrm{cik}}}}$ is the residue product ratio (RPR).

This study considered crop production data for the 2021 and 2022 calendar years from each of the 47 counties. The RPR is not constant worldwide, but is proportional to the yield in that location, which may vary depending on local factors such as plant breeding, local varieties, farming practices and prevailing environmental conditions^28,29^. Therefore, the RPR (Table S1) for each residue type from selected crops in Kenya was obtained from publications based on research in other developing countries, especially those from SSA, such as neighbouring Ethiopia. Since there were more than two RPR values reported for the same type of residue in different publications based in SSA, uncertainty in gross residue potential for every type of crop residue was represented by estimating R_cik_ using three values of RPR: the mean of all published values (RPR_1_), a low estimate based on the mean value minus one standard deviation (RPR_2_), and a high estimate (RPR_3_) based on the mean value plus one standard deviation for each residue type (Table S2 ). The value of one standard deviation was estimated using the sample standard deviation (Eq. 2):

2 $$s = \sqrt {\left( {\mathop \sum \limits_{{{\mathrm{i}} = 1}}^{{\mathrm{n}}} \left( {x_{{\mathrm{i}}} - \overline{x}} \right)/\left( {n - 1} \right)^{2} } \right)}$$

where $s$ is the sample standard deviation, $x_{{\mathrm{i}}}$ is the value of the ith RPR reported in the literature, $\overline{x}$ is the mean of reported RPR values and $n$ is the sample size.

#### Surplus crop residue potential

Surplus available residue is the fraction of gross residues that remains to be gathered after residues used for other purposes, such as livestock feeding and cooking, have been accounted for^21^. The surplus available residue of every residue type for each year from selected crops was determined using Eq. 3.

3 $${\boldsymbol{S}}_{{{\mathrm{ciky}}}} = \mathop \sum \limits_{{{\mathbf{i}} = 0}}^{{\mathbf{n}}} {\mathrm{R}}_{{{\mathrm{cikjy}}}} \times {\boldsymbol{\alpha}}_{{{\mathrm{cik}}}}$$

where for the ith type of residue from kth crop for the yth year, $S_{{{\mathrm{ciky}}}}$ is the surplus available residues (Mg), ${\mathrm{R}}_{{{\mathrm{cikjy}}}}$ is the gross available residues, and $\alpha_{{{\mathrm{cik}}}}$ is the surplus available factor (SAF).

The SAFs (Table S1) for every selected crop residue in Kenya for different types of crop residues were obtained from publications based on research in other developing countries, especially in SSA, that share similar conditions to Kenya. Since there were more than two SAF values reported for the same type of residue in different publications, uncertainty in the surplus available residues was represented by estimating S_ciky_ using three values of SAF: the mean of all published values (SAF_1_), a low estimate based on the mean value minus one standard deviation (SAF_2_), and a high estimate (SAF_3_) based on the mean value plus one standard deviation for each residue type (Table S2). The value of one standard deviation was estimated using Eq. 2 (see section “Gross crop residue potential in Kenya”), with RPR values replaced by SAF values*.*

#### Economically viable residues

Constraints on collection and transportation systems mean that not all surplus available residues can be collected and used for biochar production^27,30^. Therefore, the economically viable residues, defined as the fraction of surplus residues whose collection meets the economic feasibility criteria within a given framework, for every type of crop residue was calculated using Eq. 4.

4 $${\boldsymbol{E}}_{{{\mathrm{cikjy}}}} = \mathop \sum \limits_{{{\mathbf{i}} = 0}}^{{\mathbf{n}}} {\boldsymbol{S}}_{{{\mathrm{cikjy}}}} \times {\boldsymbol{r}}_{{{\mathrm{ci}}}}$$

where for the ith type of residue from the kth crop in the jth county for the yth year, $E_{{{\mathrm{cikjy}}}}$ is the economically viable crop residues (Mg), $S_{{{\mathrm{cikjy}}}}$ is the surplus available crop residue (Mg), and *r*_ci_ is the economically viable factor (EVF) (%).

The EVF for collecting biomass residues range from 48% (EVF_1_) to 59% (EVF_2_) of surplus residues in areas with no legal access limitations (Table S1)^27,30^. This range can be attributed to variation in access challenges due to topographical issues, suboptimal residue collection systems that lead to low residue recovery, and economically viable distance to transport the residues or the reactor^27,30^. Therefore, economically viable crop residues for each type of crop in each year were estimated separately using the minimum (EVF_1_) and maximum (EVF_2_) values quoted above for each of the nine combinations representing uncertainty in RPR and SAF. This procedure generated 18 unique treatment formulations of RPR, SAF and EVF for expressing uncertainty in the total quantity of economically viable crop residue after multiplying crop production (Q) by each factor (Table S2).

#### Crop residue density

This refers to the quantities of economic residues available in a unit area. This was estimated for each type of crop residue using Eq. 5, where the total area of each county was obtained from the Kenya National Bureau of statistics (KEBS)^31^.

5 $${\mathrm{P}}_{{{\mathrm{cikj}}}} = \frac{{{\boldsymbol{E}}_{{{\mathrm{cikjy}}}} }}{{{\boldsymbol{A}}_{{\mathrm{j}}} }}$$

where for the ith type of crop residue from the kth crop in the jth county for the yth year , ${\mathrm{P}}_{{{\mathrm{cikj}}}}$ is the economic crop residue potential density (Mg km^−2^), $E_{{{\mathrm{cikjy}}}}$ is the economical crop residue potential (Mg), $A_{{\mathrm{j}}}$ is the total area of the jth county (km^2^).

### The potential forest residues in Kenya

Forest residues were classified into logging residues, such as wood and sawdust, and other processing residues from sawmilling, veneer and plywood production.

#### The gross potential of logging and processing forest residues in Kenya

The gross potential of forest logging residues in Kenya was estimated using Eq. 6.

6 $${\mathrm{R}}_{{{\mathrm{fiy}}}} = \mathop \sum \limits_{{{\boldsymbol{i}} = 0}}^{{\boldsymbol{n}}} {\mathrm{w}}_{{\mathrm{y}}} \times \user2{ }\eta_{{\mathrm{i}}}$$

where for the ith type of residue obtained from forest logging activity in Kenya in the yth year, ${\mathrm{R}}_{{{\mathrm{fiy}}}}$ is the gross residue production (Mg), ${\mathrm{w}}_{{\mathrm{y}}}$ is the annual round wood harvested (Mg) and $\eta_{{\mathrm{i}}}$ is the residue generation ratio of the ith type of residue from logging activities.

The gross potential of processing forest residues in Kenya was estimated using Eq. 7. The residue generation ratios for logging $\left( {\eta_{{\mathrm{i}}} } \right)$ and processing $\left( {\eta_{{{\mathrm{ip}}}} } \right)$ forest operations in developing countries (Table S2) were obtained from published literature from other developing countries, especially those in SSA that share similar conditions to Kenya.

7 $${\mathrm{R}}_{{{\mathrm{pfiy}}}} = \mathop \sum \limits_{{{\boldsymbol{i}} = 0}}^{{\boldsymbol{n}}} {\mathrm{W}}_{{{\mathrm{py}}}} \times \user2{ }\eta_{{{\mathrm{ip}}}}$$

where ${\mathrm{R}}_{{{\mathrm{pfiy}}}}$ is the gross potential of processing forest residue of the ith type in the yth year (Mg), ${\mathrm{W}}_{{{\mathrm{py}}}}$ is the total amount of wood used for processing different wood products in the yth year.

#### The surplus forest residue potential in Kenya

The surplus forest residue potential in Kenya was determined using Eq. 8, with the SAFs obtained from Souza et al.^27^ and Tolessa^22^ (Table S2).

8 $${\boldsymbol{S}}_{{{\mathrm{fiy}}}} = \mathop \sum \limits_{{{\boldsymbol{i}} = 0}}^{{\boldsymbol{n}}} {\mathrm{R}}_{{{\mathrm{fiy}}}} \times \user2{ \alpha }_{{{\mathrm{fi}}}}$$

Where $S_{{{\mathrm{fiy}}}}$ is the surplus available potential of the ith type of residue from logging activities or processing wood products in Kenya for the yth year (Mg), ${\mathrm{R}}_{{{\mathrm{fiy}}}}$ is the gross potential of forest residue of ith type derived from logging activities or wood processing (Mg) in the yth year, and $\alpha$_fi_ is the SAF for the ith type of forest residue.

#### The economic forest residue potential in Kenya

The economic potential of forest residues from logging and processing activities was determined using Eq. 3 in section “The economic forest residue potential in Kenya”, with similar assumptions. Since there is only one published value for residue generation ratio and SAF available in the literature for forest residues^22^, the economically viable forest residues were estimated using two treatment formulations based on two values of EVF (48% and 59%) (Table S4).

### Mapping the distribution of crop resources for biochar production

The study used both spatial and non-spatial data to map the geographical distribution of crop residues in Kenya. Mapping the geographic distribution of forest residues was not possible because forest productivity data at county levels were unavailable. Shapefiles representing the county boundaries (polygons) in Kenya were obtained from Diva GIS vector data (https://diva-gis.org/data.html), which are freely available and accessible. Non-spatial data were the means and standard deviations (supply uncertainties) of the 18 treatment formulations of economically viable crop residues, averaged for the two calendar years (for 2021 and 2022) in each county (see section “The potential of crop residues in Kenya”). We generated the first spatial distribution map of crop residues using the grand mean of economically viable residues from the 18 treatment formulations for each county using the graduated symbology approach, based on equal intervals. The grand means of economically viable residues were classified into four groups [low (< 150,000 Mg y^−1^), medium–low (150,000–300,000 Mg y^−1^), medium–high (300,000–450,000 Mg y^−1^) and high (≥ 450,000 Mg y^−1^)]. We visualized the associated residue supply uncertainties on the distribution map, using the symbol layer’s geometry generator, set to point geometry type. The diameters of the points (circles) were adjusted to 2 mm, 4 mm, and 6 mm, to describe the three classes of residue supply uncertainties [low (< 80,000 Mg y^−1^), medium (80,000–160,000 Mg y^−1^) and high (≥ 160,000 Mg y^−1^) (see section S1.5 of the online supplementary material for the GIS code used to adjust diameters). We created the second map using the grand mean of residue densities and associated supply uncertainties (standard deviation of residue densities obtained from the 18 treatment formulations), using the same approach as for the first map. The grand means of residue densities were classified into four groups [low (< 20 Mg y^−1^ km^−2^), medium–low (20 -100 Mg y^−1^ km^−2^), medium–high (100–160 Mg y^−1^ km^−2^), and high (≥ 160 Mg y^−1^ km^−2^)]. The uncertainties in residue supply based on residue densities were categorized into three groups [low (< 50 Mg y^−1^ km^−2^), medium (50—100 Mg y^−1^ km^−2^) and high (≥ 100 Mg y^−1^ km^−2^)].

### Data analysis

The statistical analysis was conducted using R software version R v 4.4.3 (see Section S1.5 for R code). The study used repeated measure statistical techniques, as crop and forest productivity were measured in Kenya across two consecutive years (2021 and 2022), where factors that influenced productivity in 2021 were also likely present in 2022. Non-parametric data analysis techniques were used because the Shapiro–Wilk test showed that residuals were not normally distributed (*p* < 0.05). Non-parametric Wilcoxon signed-rank tests were used to analyse the statistical differences in the crop and forest productivity between the two years. A Morris sensitivity analysis was used as a screening technique to determine which input factors (crop and forest productivity, RPR, SAF and EVF) had most influence on the economically viable crop and forest residues for biochar production^32^. The results are visualized as a plot of absolute main effect (μ ∗) on the y-axis against interactions (σ) on the x-axis. The most influential variables occupy the top-left quadrant, while variables with least influence are in the bottom-right quadrant^35^.

## Results

### Crop and forest productivity in Kenya

Total crop productivity was 2.98 × 10^7^ Mg in 2021 and 2.99 × 10^7^ Mg in 2022 (Table S5), while wood production was 9.33 × 10^5^ Mg in 2021 and 9.98 × 10^5^ Mg in 2022 (Table S6). The Wilcoxon signed-rank tests showed that these increments in crop productivity (V = 314.5, *p* = 0.087) and forest productivity (V = 13.00, *p* = 0.152) were not statistically significant between years, suggesting a stable supply of economically viable crop and forest residues for biochar production. This counters the hypothesis that instability of the interannual supply will reduce economic viability of biochar production plants. However, these findings should be interpreted with caution as two-year data may not reflect long-term trends. Since the variability in crop production between the two years was not statistically significant, estimation of crop and forest residues was estimated from the mean across both years.

### Crop residue potential in Kenya and their sensitivity

Kenya produces (5.02–23.61) × 10^6^ Mg y^−1^ of economically viable crop residues for biochar production, depending on the treatment formulation of RPR, SAF and EVF used for estimation (Table 1). The data in Table 1 suggest that RPRs, SAFs and EVFs are critical determinants of economically viable crop residues available and their corresponding residue densities for biochar production. A Morris sensitivity analysis suggested that crop productivity was the primary driver of crop residues, followed by SAF and RPR, while the EVF was the least influential variable (Figure S1). These findings suggest that increasing crop productivity would increase the amount of economically viable crop residues for biochar production, despite their competing uses.

**Table 1:** Economically viable crop residues for biochar production in Kenya based on different treatment formulations of residue to product ratio (RPR), surplus available factor (SAF) and economically viable factor (EVF).

RPR estimates	SAF estimates	EVF (%)	Mean ± SD	
			Economically viable residues (1 × 10^6^ Mg y^−1^)	Densities of economically viable residues (1 × 10^3^ Mg y^−1^)
Low	Low	48	5.02 ± 0.09	1.85 ± 0.09
Low	Low	59	5.03 ± 0.09	1.85 ± 0.09
Low	Mean	48	9.22 ± 0.09	3.57 ± 0.12
Low	Mean	59	9.22 ± 0.09	3.57 ± 0.12
Low	High	48	11.07 ± 0.25	4.34 ± 0.23
Low	High	59	11.07 ± 0.25	4.34 ± 0.23
Mean	Low	48	7.84 ± 0.08	2.99 ± 0.11
Mean	Low	59	7.84 ± 0.08	2.99 ± 0.11
Mean	Mean	48	14.44 ± 0.16	5.67 ± 0.30
Mean	Mean	59	14.44 ± 0.16	5.69 ± 0.30
Mean	High	48	17.27 ± 0.41	6.87 ± 0.37
Mean	High	59	17.27 ± 0.41	6.87 ± 0.37
High	Low	48	10.58 ± 0.22	4.09 ± 0.21
High	Low	59	10.58 ± 0.22	4.09 ± 0.21
High	Mean	48	17.52 ± 2.77	6.99 ± 1.33
High	Mean	59	17.52 ± 2.77	6.99 ± 1.33
High	High	48	23.53 ± 0.54	9.39 ± 0.50
High	High	59	23.61 ± 0.54	9.39 ± 0.50

The values in Table 1 are averaged across years, which was not significant.

SD is the standard deviation.

In contrast, SAFs and RPRs were primary drivers of crop residues densities, followed by crop production, while the EVF was again the least influential variable (Figure S2). In contrast, SAFs and RPRs were primary drivers of crop residues densities, followed by crop production, while the EVF was again the least influential variable (Figure S2).

These findings suggest that the density of crop residues for biochar production would decrease with increasing competing uses of residues and decreasing RPRs, suggesting that the density of crop residues for biochar production would decrease with an increase in the intensity of competition for residues or with decreases in RPRs.

### Forest residue potential in Kenya

Kenya could produce (1.48−1.8) × 10^5^ Mg y^−1^ of forest residues depending on the applied EVF, which ranges from 48 to 59%. Forest residues originate predominantly from solid wood and sawdust [(3.24−4.21) × 10^4^ Mg y^−1^] from logging and processing locally harvested logs (Table 2). These findings could be attributed to higher domestic throughput from locally harvested logs than imported logs, depicting a robust domestic supply chain of forest residues that is insulated from import fluctuations. Morris’s sensitivity analysis showed that forest productivity was the primary driver of forest residues, with SAFs and RPRs having equal effects, then EVF recorded least effect (Figure S3). Therefore, increasing sustainable harvesting of local forests through enhanced tree planting initiatives could ensure a sustainable supply of forests residues for biochar production.

**Table 2:** Economically viable forest residues for biochar production in Kenya based on different levels of economically viable factor.

Type of wood	Activity	Type of residue	Economically viable factor used	Economically viable forest residues (mean ± SD 1 × 10^4^ Mg y^−1^)
*Harvested in Kenya*				
Sawlogs, veneer logs, pulpwood, roundwood and split wood and sawn wood	Logging	Dust	48%	1.71 ± 0
			59%	2.11 ± 0.01
		Solid wood	48%	3.42 ± 0
			59%	**4.21 ± 0.03**
	Processing	Sawdust	48%	3.24 ± 0
			59%	**4.00 ± 0.03**
		Solid wood	48%	2.39 ± 0
			59%	2.95 ± 0.02
*Imported into Kenya*				
Industrial roundwood	Processing	Sawdust	48%	1.15 ± 0.19
			59%	1.41 ± 0.23
		Solid wood	48%	0.85 ± 0.14
			59%	1.04 ± 0.17
Sawn wood	Processing	Sawdust	48%	0.87 ± 0.10
			59%	1.07 ± 0.13
		Solid wood	48%	1.18 ± 0.14
			59%	1.45 ± 0.17

The values in the Table are averaged across years because the difference between the two years was not significant.

### Spatial distribution of economically available crop residues in Kenya

**Fig. 1:**
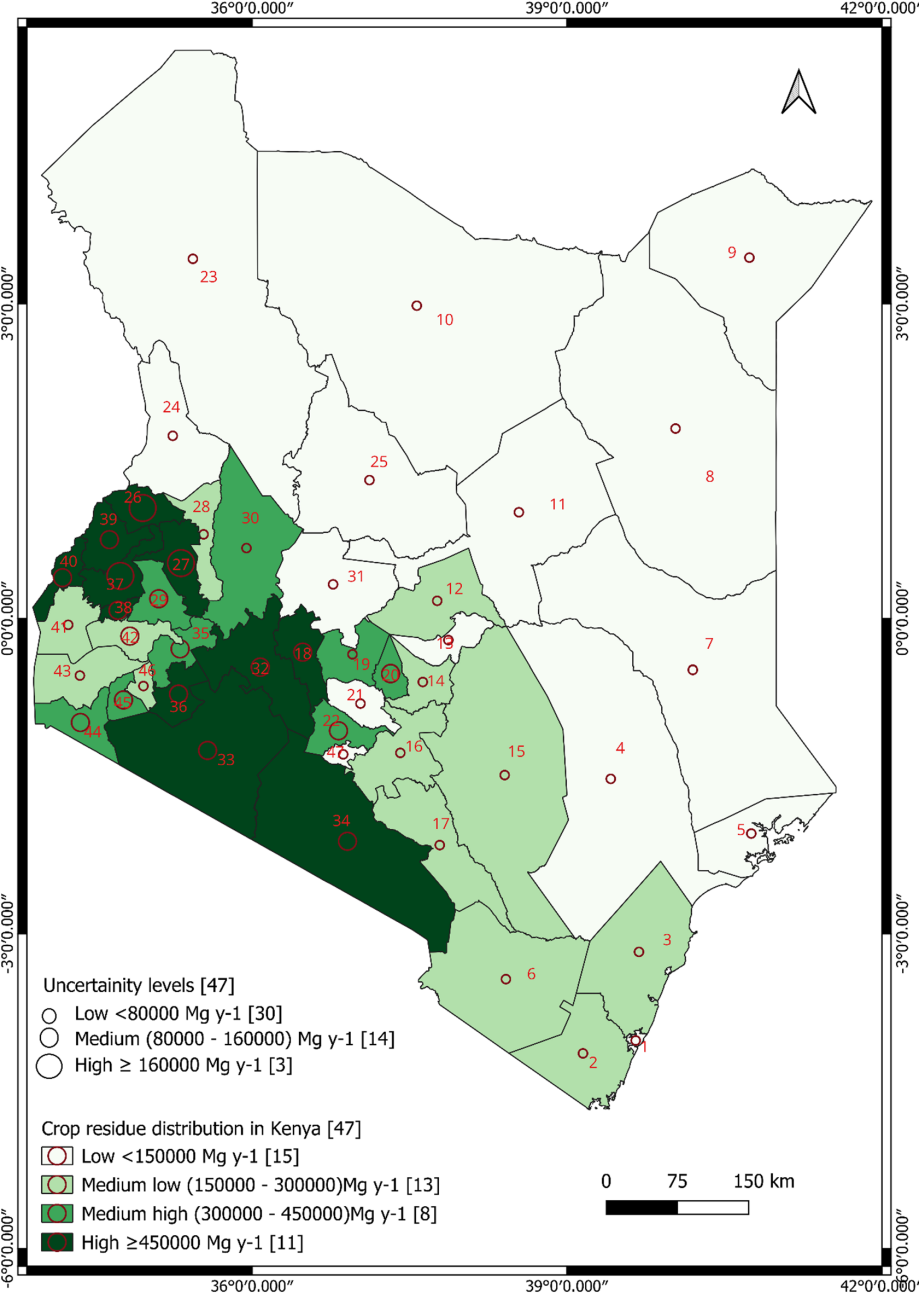
Classification of crop residue distribution across the 47 counties of Kenya based on the averaged 2021 and 2022 crop production data. Map created by author using QGIS (v. 3.34.14; https://www.qgis.org/de/site/). Shapefile data provided by DIVA-GIS (https://diva-gis.org/data.html).

**Fig. 2:**
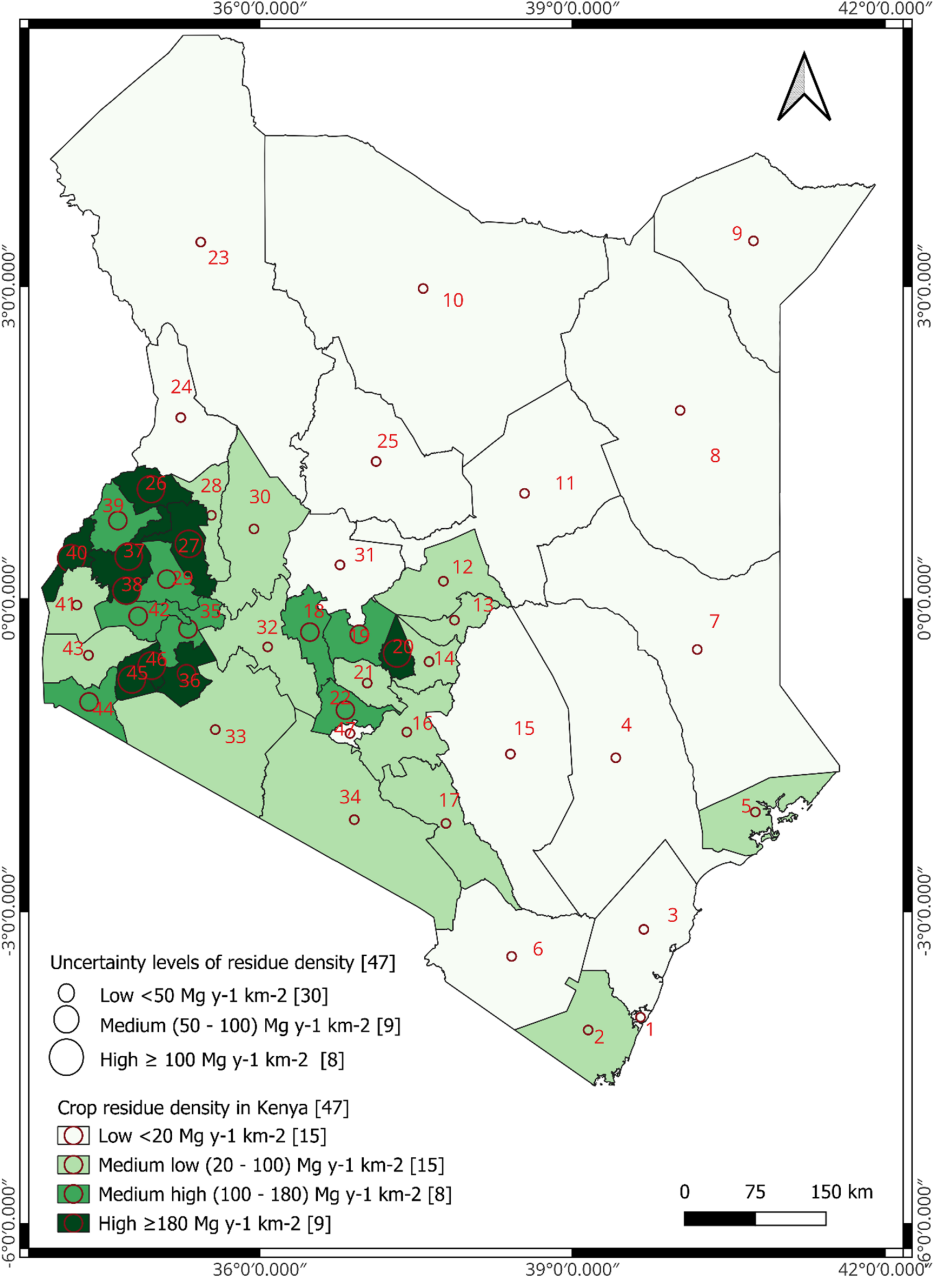
Classification of crop residue density distribution across the 47 counties of Kenya based on the averaged 2021 and 2022 crop production data. Map created by author using QGIS (v. 3.34.14; https://www.qgis.org/de/site/). Shapefile data provided by DIVA-GIS (https://diva-gis.org/data.html).

The spatial distribution of economically viable crop residues for biochar production across Kenya was non-uniform, with significant concentrations in the western, central and southern parts of the country (Figs. 1 and 2 with labels defined in Table S7). The results indicate that 11 counties (Bungoma, Bomet, Busia, Kajiado, Kakamega, Nakuru, Narok, Nyandarua, Trans-Nzoia, Uasin Gishu, Vihiga) recorded very high amounts of economically viable crop residues for biochar production (≥ 4.5 × 10^5^ Mg y^−1^) (Fig. 1). However, none of them recorded the required high residue densities (≥ 300 Mg km^−2^) with low supply uncertainty (< 50 Mg km^−2^) (Figs. 1 and 2). Kakamega, Trans-Nzoia and Uasin Gishu recorded the required high densities but had a high uncertainty in supply (≥ 100 Mg km^−2^). Conversely, Kajiado, Nakuru and Narok recorded lower densities than required but with low supply uncertainty. Among the eight counties that recorded medium–high amounts of crop residues, none recorded high residue densities (Fig. 1). Medium low (1.5−3.0 × 10^5^ Mg y^−1^) and low (< 1.5 × 10^5^ Mg y^−1^) amounts of residues were recorded in a total of 19 counties, all recording lower densities than required but with low supply uncertainties (Fig. 1). This non-uniform distribution is expected to be associated with bioclimatic conditions, such as annual precipitation, supporting the hypothesis that residues are characterized by spatial heterogeneity at county level, reflecting bioclimatic variations. Again, this spatial distribution should be interpreted with caution, given that it is based on only two calendar years of data.

### Main crop residues available for biochar production in each county

**Fig. 3:**
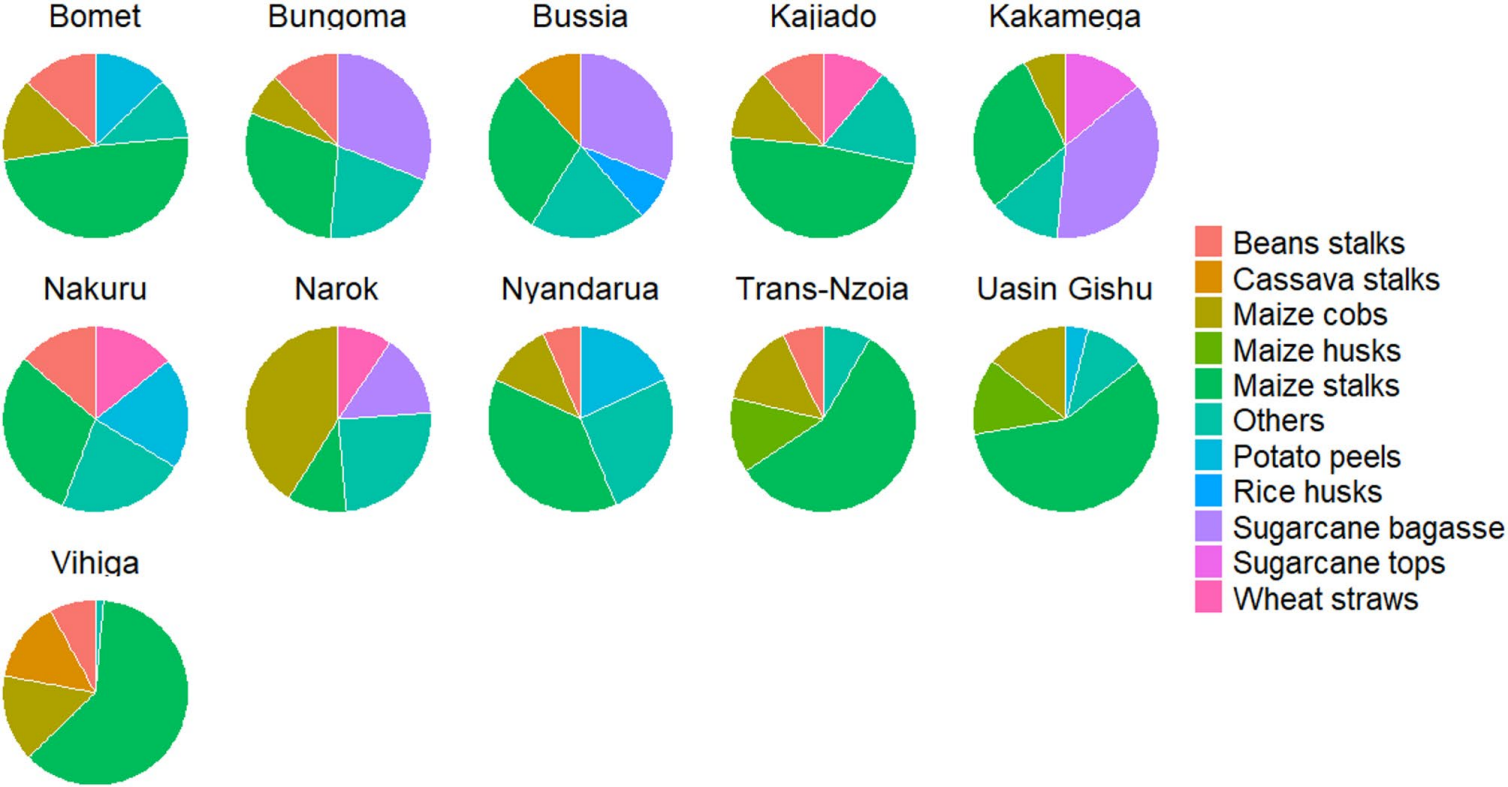
Types of crop residues dominating in counties with high amounts of economically viable crop residues available for biochar production.

**Fig. 4:**
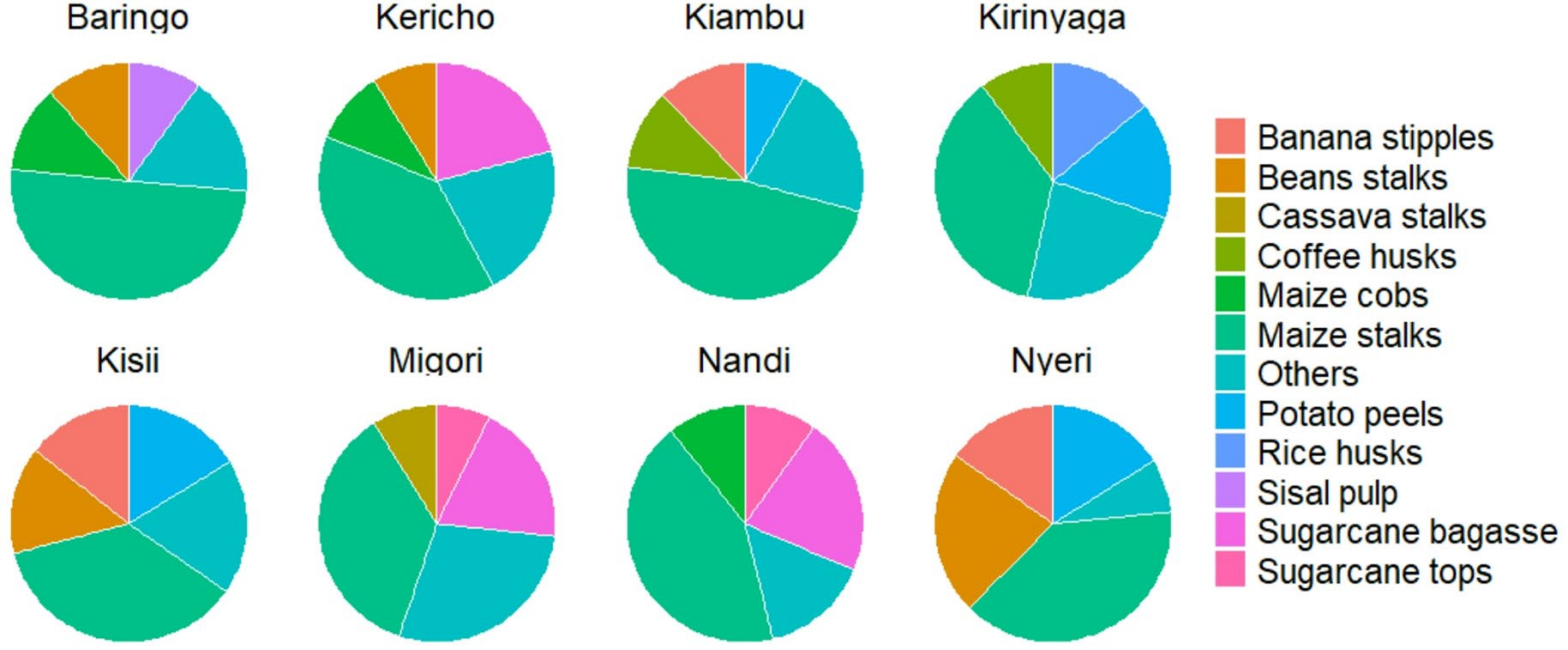
Types of crop residues dominating in counties with medium high amounts of economically viable crop residues available for biochar production.

**Fig. 5:**
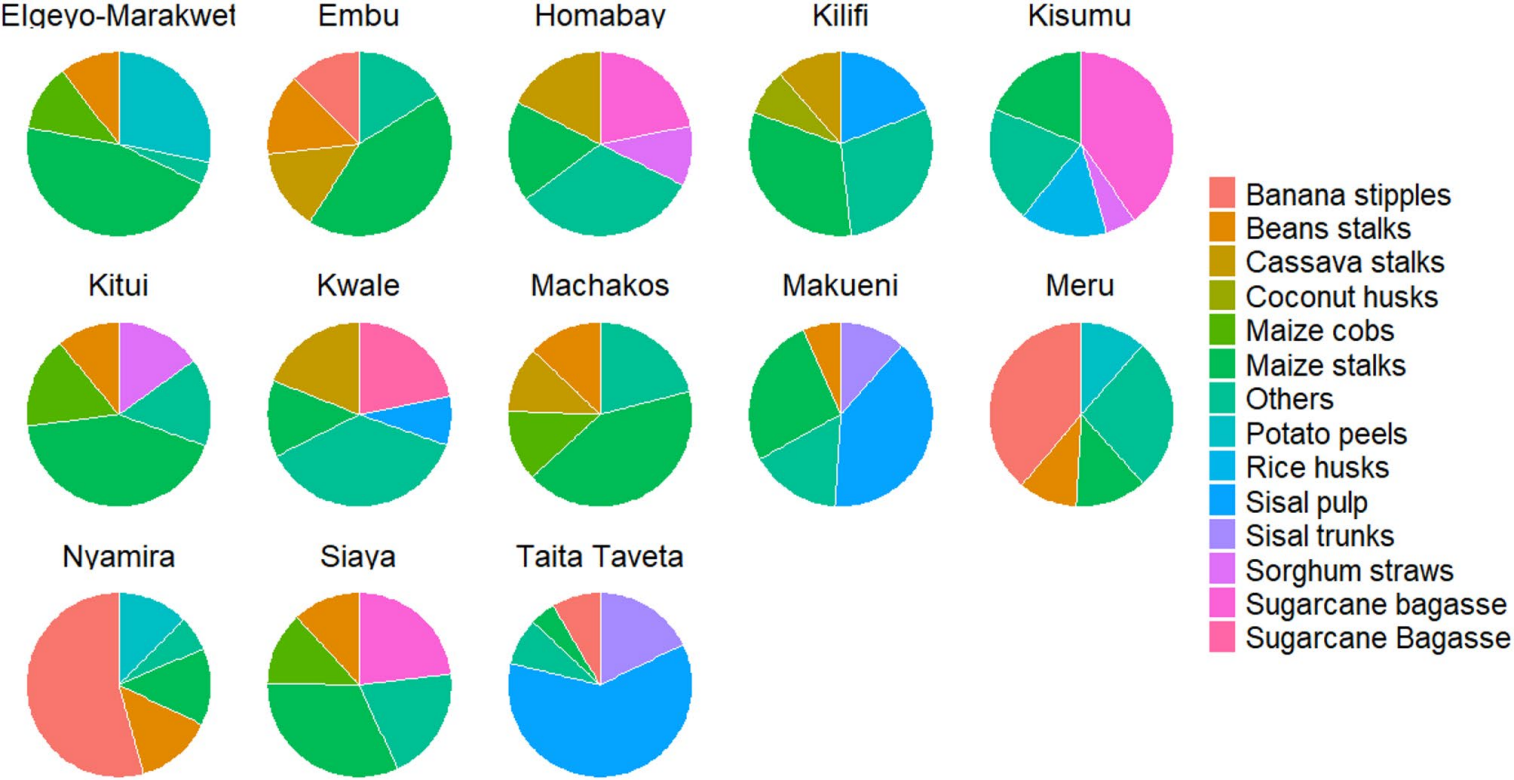
Types of crop residues dominating in counties with medium low amounts of economically viable crop residues available for biochar production.

**Fig. 6:**
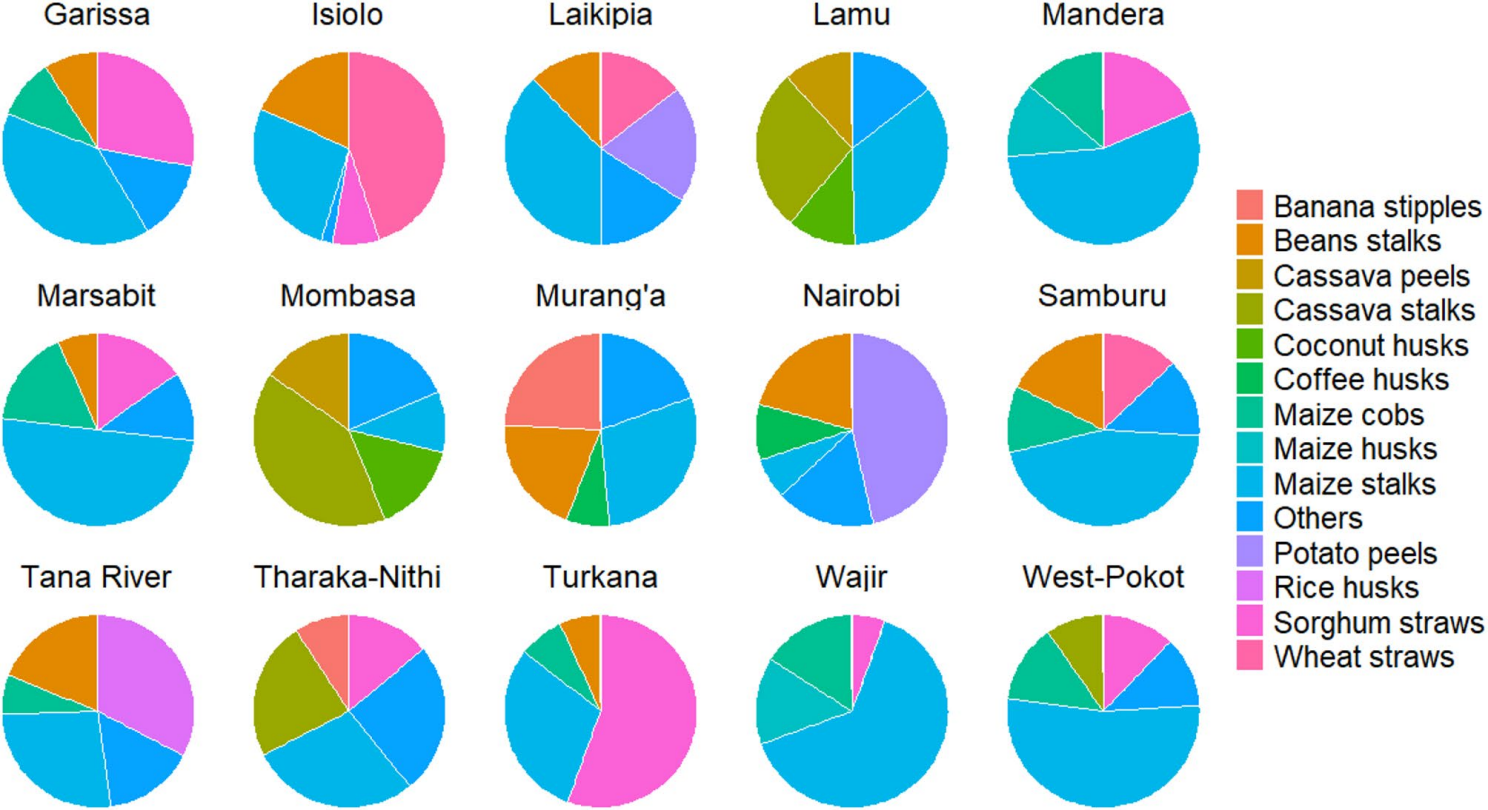
Types of crop residues dominating in counties with low amounts of economically viable crop residues available for biochar production.

The most abundant types of crop residue differed between counties, but maize stalks were the most prevalent type listed in all 47 counties (Table S8). Maize is a staple food in Kenya and so has undergone intensive selective breeding to enhance its resilience across a wide range of bioclimatic conditions. Some crop residues, such as sisal pulp, were only found in five counties located in the semi-arid regions, namely Taita Taveta (61.01%), Makueni (50.50%), Kilifi (18.94%), Baringo (10.05%) and Kwale (8.53%) (Figs. 3, 4, 5 and 6, and Table S8). Only two counties, Homabay and Kwale, were dominated by crop residues in the “others” category (Fig. 5). These findings suggest that Kenyan counties have diverse crop residues resulting from cultivation of different crops depending on bioclimatic conditions.

## Discussion

This study has comprehensively assessed available crop and forest residues for biochar production, and their spatial distribution across the whole of Kenya, to foster development of residue processing plants. This section interprets the findings in the context of existing literature, and presents the challenges, limitations, novelty and global implications of the study.

### Crop and forest residues for biochar production

The results indicate that crop and forest productivity in 2021 and 2022 were not statistically different. This could be because the two-year period was too short to observe directional changes in crop production^33^, and/or because climatic conditions were similar between these years. Hence these estimates do not fully capture long-term averages that are subject to the interannual variability. Nevertheless, these data suggest that annual production is relatively consistent between years, even during the global Covid-19 pandemic. For example, despite the imposition of partial Covid-19 lock-downs in Kenya in March 2021, which resulted in travel restrictions between counties, production in the agricultural sector grew by 6.4%, demonstrating the robustness and resilience of the agricultural sector in Kenya to extreme non-climatic events^34^. This insight is critical to crisis preparedness, as it sets a new baseline for acknowledging the systematic resilience in policy and decision making, other than by considering the long-term averages in isolation.

We estimate that Kenya could produce (0.5−2.4) × 10^7^ Mg y^−1^ of crop residues and (1.48−1.8) × 10^5^ Mg y^−1^ of forest residues for production of economically viable biochar. However, the actual potential could be higher as the study considered only crop and forest productivity data from government ministries, FAOSTAT and other national reports, which only consider production that goes through formal and commercial sectors^36,37^. Much of crop and forest production in SSA, including Kenya, is conducted informally, and never recorded^38^. This unrecorded production arises because smallholder stakeholders in the agriculture and forest sectors do not see the importance of formal systems, want to evade taxes charged in formal systems, or are not ready or able to keep proper records^39^. Therefore, to enhance formalization of smallholder entrepreneurs, there is a need to implement favourable policies, such as a zero-rate tax regime, to reduce book-keeping requirements and to conduct capacity building that highlights the benefits of formalization for smallholder enterprises.

Morris sensitivity analysis showed that the amounts of economically viable crop and forest residues were primarily influenced by the crop and forest production, with the EVF having the least effect. Therefore, on-going efforts to improve crop productivity, such as farmer training in better agronomic practices, irrigation and adoption of climate-smart agriculture, will also ensure a sustainable supply of residues for biochar production^9,40^. Furthermore, on-going propagation of trees, such as *Melia volkansii* in drylands for commercial forestry, will help to secure a sustainable supply of forest residues^41^. Conversely, increasing alternative uses of crop residues, such as livestock feeding, mulching and use of residues for cooking and heating energy, could reduce the availability of residues for biochar production^38^. Furthermore, modern plant breeding approaches that emphasise increasing the yield harvest index without significant increase in total crop biomass^28,29^, would also reduce the amounts of available residues for biochar production. However, efficient production of quality biochar for soil amendment could increase total crop biomass production^1,2^, and provide an alternative to using raw residues^42^, thereby reducing pressure on crop residues. Caution should be exercised when improving the yield efficiency of biochar production, however, as the same factors that might be tuned to improve yield, such as heating rate and pyrolysis temperature, also affect the quality of biochar^43^. In addition, use of top-lit updraft (TLUD) cookstoves could also increase biochar production, because these devices produce both biochar and energy for the household, which reflects the important role of crop residues for energy production in SSA^5^. Nevertheless, most simple and affordable TLUD cookstoves in SSA generate very little biochar (0.3–1 kg), making them practical for small-scale uses only^44^. This suggests the need to enhance the production capacity of TLUDs while avoiding increases to their cost. Therefore, our results suggest that Kenya has a sustainable supply of feedstock for running biochar production facilities, but there is a need to upgrade existing production technologies in terms of efficiency and capacity.

The findings that crop residues are heavily concentrated in the western and south-central parts of Kenya can be attributed to the more suitable soils and climatic conditions of these regions, such as higher mean annual rainfall and temperature, which favour crop production^45,46^. Locating biochar production facilities in regions that have at least moderate concentrations of residues reduces the cost of collecting and transporting residues to centralized facilities^47^, which accounts for about 21% of biochar production costs in countries, such as Canada^48^. While some counties were classified as having very high amounts of economically available crop residues for biochar production (e.g. Nakuru, Fig. 1), none of them had the required combination of high residue density and low supply uncertainty, as determined by variation in crop production, RPR, SAF and EVF) (Fig. 2). This is because these counties occupy a larger geographical area than others^31^, and available residue density is primarily driven by RPR and SAF (Figure S2). Therefore, in counties with high amounts but low densities of residues and low uncertainty, such as Nakuru, collection and transportation of residues to centralized production units may be costly, leading to a higher carbon footprint and requiring complex supply chain coordination^49,50^. On the other hand, in counties such as Trans-Nzoia, with high amounts and densities of residues but high supply uncertainties, the quantity of residues available in a unit area at a particular time may be unpredictable, increasing the possibility that residues will have to be collected and transported over greater distances^49–51^. Therefore, identifying the optimal location of a biochar production facility in Kenya requires a trade-off between choosing areas with high residue densities or those with low supply uncertainties. Alternatively, the national and county governments could formulate policies that promote decentralized and mobile micro-pyrolysis units that operate economically at small scale, to compliment large, centralized plants. This could include offering grants or tax credits to purchase efficient pyrolysis units through community cooperative ownership, and subsidies for feedstock stockpiling facilities to handle initial feedstock pre-treatments, such as drying and compaction to increase the storage time^52^.

The northern, eastern and parts of the central regions of Kenya recorded low amounts of crop residues for biochar production with low residue densities. This is driven by their less suitable climates for crop growth and poor soil conditions^53^, resulting in low agricultural and forestry activities and competing uses for the residues. These regions are, however, heavily infested by invasive non-native species that could be used for biochar production. For example, *Prosopis juliflora* occupies over 1.5 × 10^6^ ha, in arid and semi-arid regions of Kenya, accounting for over 50% of these lands^54^. Exploitation of invasive non-native species for biochar could be incorporated into the control strategies for these species and help to mitigate their negative economic effects on agricultural productivity and biodiversity^55^.

Maize stalks were the most prevalent type of crop residue listed in all counties. This is because maize is the main crop grown in Kenya, especially among the smallholder farmers that account for about 75% of overall production^16,17,56^. Maize grows well in Kenya because available hybrids are adaptable to a wide range of agro-ecological zones^16,57^, compared to other crops, such as sisal (*Agave sisalana*), that only grow in hot, arid and semi-arid regions characterised by sandy soils and mean annual rainfall less than 600 mm^58^. The diversity of crop residues in each county moderates the effect of seasonal fluctuation in feedstock availability for optimal production and running of the facility^47^. This indicates a need to design biochar production kilns that are sufficiently flexible to accommodate a variety of feedstocks^44^. Different feedstocks have variable optimal pyrolysis parameters, such as maximum pyrolysis temperature, heating rates and pressures, which can be regulated through installation of tighter control mechanisms on the kiln design^43,44^. Furthermore, sawdust is the leading forest residue as chainsaws, with a conversion rate of raw logs to timber of < 30%, are widely used in saw milling due to their portability, relative affordability and ease of operation, which is not limited by terrain^59^. Despite their potential for multiple uses, including bioenergy, mulching and fodder^42^, maize stalks and sawdust still dominate the supply chain of economic residues for potential biochar production. Maize residues are in most cases burnt in the field to prepare land for subsequent planting, control pests and diseases and avoid the costs of collection and transportation, and sawdust is piled on site at sawmills^19^. Incineration of these residues leads to loss of their embedded nutrients and carbon and increases the chances of unwanted fires in sawmills^44^.

### The challenges, limitations, novelty and relevance of the study

While the study provides a comprehensive assessment of crop and forest residues in Kenya, the findings should be interpreted within the context of inherent challenges and limitations. This is because the challenges encountered during data collection may have affected the estimates of processed residues available for biochar production in each county. For example, we were unable to obtain important data on the cross-border flows of agri-food products among counties. According to Otieno^60^, both formal and informal trade in agricultural products occurs between counties in Kenya, and between Kenya and its neighbouring countries. This implies that after harvesting, some residues, such as maize husks and bagasse from processing activities, and others, such as coconut husks and banana peels that are generated while using the product, are transported from their counties of origin to other counties or countries. Although we did not account for these flows of residues in our estimates, over 75% of farmers in Kenya are subsistence smallholders^56^ and so, failure to account for these flows of residues would have had limited impact on the estimates. The exception is sugarcane bagasse residue, which is generated in milling factories after transporting the canes.

Another critical challenge was establishing appropriate values for RPR and SAF to reflect the Kenyan context. This study used information from other low-income countries, such as Ethiopia, in estimating economical residue potential for biochar production in Kenya. These factors may vary among countries and regions depending on improvements associated with crop breeding, the level of technology employed in harvesting and processing, weather conditions and the socioeconomic status of the community^28,29^. Published studies, such as Kimutai et al.^16^, provide generalized values, but these ratios may vary among households within the same locality, with low and high social class households having different values. The use of conversion ratios (RPR and SAF) derived from published literature introduces uncertainty in our estimates of residue availability. This is because RPRs and SAFs are context specific and potentially variable within the region, so, values appropriate to the local practices and varieties of Kenya could be different from published values from other regions or countries. We attempted to mitigate this uncertainty by calculating low, medium and high estimates based on the range of published values of RPR and SAF, and then combining them factorially to determine the range of likely estimates based on this variation. This approach enhanced the realism of the results by simulating a range of possible scenarios for our Kenyan case study.

The fundamental limitation of this assessment is its reliance on secondary data from the Ministry of Agriculture and Livestock Development over a two-year period. While use of secondary data allows a broader study area, we had no control over the protocol or inherent sampling used in primary data collection by the ministry. Also, the use of two-year data may not account for long-term interannual variations, seasonal shifts or policy changes at the national and county levels. Therefore, while the study provides a robust assessment of viable crop and forest residues for biochar production in Kenya, they should be interpreted as indicative rather than absolute. Therefore, future research should consider direct and longitudinal field measurement of crop and forest productivity to enhance the precision of residue inventories. Such efforts would further determine local-based (Kenyan-based) conversion factors (RPRs, SAFs and EVFs).

Despite the challenges and limitations, this study provides a novel multilayered analytical approach that can be used in any geographical context. We used a resource-based residue assessment approach using the quantity of harvest, RPRs, SAFs and EVFs, adapted from existing literature. However, we incorporated a new approach to evaluating uncertainty by using the mean value, a low estimate based on the mean value minus one standard deviation, and a high estimate based on the mean value plus one standard deviation of RPRs and SAFs for every type of residue. Furthermore, the use of the Morris sensitivity analysis provides methodological rigor that screens primary drivers of economically viable crop and forest residues. We believe this provides insightful information to policy- and decision-makers on factors to focus on when assessing available residues for biochar production. When mapping the spatial distribution of available crop residues, we included the supply uncertainties to address the operational realities, other than hypothetical mean values only. The approaches [(mean value, low and upper estimates), Morris sensitivity analysis and inclusion of supply uncertainties] departs from existing methodological conventions seen in other studies^14,16–19^. This study, therefore, introduces a higher degree of methodological rigor, transitioning from simple residue estimation and mapping to a comprehensive evaluation and visualization that includes the supply uncertainties.

The methodology used in our study is inherently universal, thus provides a robust framework that can be replicated to assess available economically viable crop and forest residues in any geographical context. Kenya has diverse climates and is inhabited by people of a wide range of socio-economic status. Therefore, our methods provide a novel framework for predictive mapping of spatial heterogeneity in residue distributions in other regions of SSA and beyond that experience similar diversity in bioclimatic conditions and human societies.

## Conclusions and recommendations

Based on data from the 2021 and 2022 calendar years, this study finds a diverse range of residue types across Kenya, with highest abundance in the western, central and southern counties. These residue quantities are primarily driven by crop and forest production, RPRs and SAFs. Such findings suggest a need for improvements in the production capacity and yield efficiency of existing biochar production technologies, without compromising the quality of biochar and affordability of the technology. This approach could in turn optimize utilization of unused residues for soil amendment and fuel briquetting for climate change mitigation and adaptation. Siting of biochar production facilities in Kenya requires a trade-off between high residue density and low uncertainty in supply quantities, as no county satisfies the combined requirements of high quantity, high density and low uncertainty in the availability of crop residue.

We advocate for widespread adoption of this approach for mapping and estimation of biomass residues in other settings, especially for countries within SSA, and for further research to derive locally applicable values of the conversion factors that have a strong influence over these estimates. We also recommend that national and county governments formulate favourable policies that promote decentralized and mobile small-scale production units owned by community cooperatives through incentives, such as grants or tax credits, to purchase efficient pyrolysis technologies. They could also offer subsidies to develop feedstock stockpiling facilities to buffer supply uncertainties. The volume of stockpiling facilities needed can be estimated from the uncertainty in the supply. This study could not map forest residues in Kenya due to inadequate availability of data to enable spatial mapping. We therefore recommend that the Kenya Forest Service (KFS) develops an online database of wood volumes harvested, exported from, and imported into, each county. Such data would permit greater resolution of our recommendations for locating new biochar production infrastructure.

## Supplementary Information

Supplementary Information 1.

Supplementary Information 2.

## Data Availability

The datasets used or analysed during the current study are attached as a supplementary material in excel file.
