# Supplementary material for: Availability and spatial distribution of crop and forest biomass residues for biochar production in Kenya
Source: Sci Rep. 2026 Mar 2;16:11764. doi: 10.1038/s41598-026-42350-0 (PMC13066414; doi:10.1038/s41598-026-42350-0)
Supplement: Supplementary file 1 — Supplementary Information 1. [file 41598_2026_42350_MOESM1_ESM.docx]

**Availability and spatial distribution of crop and forest biomass residues for biochar production in Kenya**

**Timothy Namaswa^1,2, 3,4^, David F.R.P. Burslem^1, 3^, Jo Smith^1^, Waheed Afzal^4^, Jennifer Wardle^1^, Nellie Oduor^2^, Leonard Kubok^5^, George Muthike^2^ Faith Malei^2^**

^1^ School of Biological Sciences, University of Aberdeen, St Machar Drive, Aberdeen, AB24 3UU, Scotland, UK

^2^ Kenya Forestry Research Institute, P. O. Box 64636–00620, Nairobi, Kenya

^3^ Interdisciplinary Institute, University of Aberdeen, King's College, Aberdeen, Scotland, UK AB24 3FX

^4^ School of Engineering, University of Aberdeen, Fraser Noble Building, Aberdeen, Scotland, UK AB24 3UE

^5^ Ministry of Agriculture and Livestock Development KE, Cathedral Road Nairobi, P. O. Box 30028-00100, Nairobi, Kenya

**Supplementary material**

**Section S1: Supplementary materials for the materials and methods section**

**Section S1.1. Residue conversion factors for crop residues**

**Table S1: The ratio factors for estimating crop residues from total crop productivity for biochar production**.

This table provides information on moisture content, residue product ratios (RPR), surplus available factors (SAF) and economically available residue factors of crop residues from literature published in developing countries with a specific focus in Sub-Saharan Africa. It also shows their means and standard deviations

| **Type of crop** | **Type of residues** | **Moisture content (w/w) (%)** | **RPR** | **Mean RPR and standard deviations** | **SAF** | **Mean SAF and standard deviations** | **Recoverable fraction** |
| --- | --- | --- | --- | --- | --- | --- | --- |
| Green Bananas | Peelings | 84.50 [1] | 0.34,0.3, 0.4 [12,13,14] | 0.35±0.05 | 0.8, 0.3 [16,22] | 0.55±0.35 | 0.48, 0.59 [18,27] |
|  | Stipple | 90.75 [1] | 3.7, 5.6 4.46, 5 [12,13,15,16,17] | 4.69±0.81 | 0.8, 0.3 [16,22] | 0.55±0.35 | 0.48, 0.59 [18,27] |
| Beans | Stalks | 20.0 [2] | 2.3, 2.66, 2.01, 2.13 ,2.3 [15,16,18,19,20,21,22,23] | 2.28±0.25 | 0.5, 0.3, 0.8, 0.5, 0.3,0.7 [16,18,19,20,21,22] | 0.52±0.21 | 0.48, 0.59 [18,27] |
| Cassava | Peelings | 66.08 [3] | 0.65, 0.64, 1.11,0.91 [15,16,18,20] | 0.83±0.23 | 0.4, 0.5,0.3 [16,18,20] | 0.4±0.1 | 0.48, 0.59 [18,27] |
|  | Stalks | 24.93 [4] | 2, 1 [9,16,21] | 1.5±0.71 | 0.5, 0.3 [16,21] | 0.4±0.14 | 0.48, 0.59 [18,27] |
| Coconut | Fronds | 30.0 [5] | 0.47 [12] | 0.47±0.0 | 0.47 [12] | 0.47±0.0 | 0.48, 0.59 [18,27] |
|  | Husks | 26.0 [6] | 0.84, 1.01,0.84 [15,16,18,20,21] | 0.90±0.1 | 0.9, 1,0.5 [16,18,20,21] | 0.8±0.27 | 0.48, 0.59 [18,27] |
|  | Shell | 26.0 [6] | 0.41, 0.42, 0.21 [15,20,21] | 0.345±0.12 | 0.3 [20,21] | 0.3±0.00 | 0.48, 0.59 [18,27] |
| Coffee | Husks | 13.3 [7] | 0.59, 2.1, 1,0.59, 1.16,0.59,1.88, [13,15,16,18,19,20,21,22] | 1.13±0.63 | 0.5, 1, 0.58, 0.4, 0.3,1 [16,19,20,21,22] | 0.63±0.30 | 0.48, 0.59 [18,27] |
| Groundnuts | Shell/husks | 11.5 [8] | 0.79, 1.25,1.2 [15,16,21,23] | 1.08±0.25 | 0.3, 0.3,0.9 [16,21,22] | 0.5±0.35 | 0.48, 0.59 [18,27] |
| Maize | Cobs | 15.0 [9] | 0.27, 0.273, 0.57, 0.35, 0.4,0.3 [9,12,13,15,19,21,23] | 0.36±0.12 | 1, 0.40,1 [19,21,22] | 0.8±0.35 | 0.48, 0.59 [18,27] |
|  | Stalks | 15.0 [9] | 1.68, 2, 2.44, 1.53, 1.5, 2.14, 1.93, 4.33,1.7, 2 [9,13,15,16,18,19,20,21,22,23] | 2.13±0.83 | 0.4, 0.8, 0.5, 0.4, 0.3,0.8 [16,18,19,20,21,22] | 0.53±0.22 | 0.48, 0.59 [18,27] |
|  | Husks | 11.11 [9] | 0.6, 0.25, 0.2, 0.40, 0.3, 0.2, [9,13,15,16,19,22] | 0.33±0.16 | 1, 0.3,1 [16,19,22] | 0.77±0.40 | 0.48, 0.59 [18,27] |
| Potatoes | Peels | 77.0 [10] | 1.14, 1.14,0.8 [15,16,22] | 1.10±0.2 | 1 [16,22] | 1±0.0 | 0.48, 0.59 [18,27] |
| Rice | Straws | 15.0 [9] | 1.55, 1.75, 2.18, 1.54, 3.96, 1.1, 3.96,1.5,1.5 [9,15,16,18,19,20,21,22,23] | 2.12±1.08 | 0.4, 0.8, 0.5, 0.55, 0.4,0.5 [16,18,19,20,21,22] | 0.53±0.15 | 0.48, 0.59 [18,27] |
|  | Husks | 15.0 [9] | 0.275, 0.26, 0.26,0.23, 0.27, 0.35, 0.3,0.2 [13,15,16,19,20,21,22,23] | 0.27±0.05 | 0.83, 0.5, 0.55,0.4, 0.8 [16,19,20,21,22] | 0.62±0.19 | 0.48, 0.59 [18,27] |
| Sisal, raw | Pulp | 26.11 [11] | 24 [24] | 24±0.00 | 0.9 [25] | 0.9±0.00 | 0.48, 0.59 [18,27] |
|  | Bogas/trunk | 26.11 [11] | 7 [24] | 7 ±0.00 | 0.9 [25] | 0.9±0.00 | 0.48, 0.59 [18,27] |
| Sorghum | Straws | 15.0 [9] | 1.9, 1.75, 4.13, 1.9, 1.25, 7.4 [9,15,16,18,19,20] | 3.06±2.35 | 0.8, 0.8, 0.25, 0.3 [16,18,19,20] | 0.54±0.30 | 0.48, 0.59 [18,27] |
| Sugar cane | Tops/leaves | 70 [26] | 0.32, 0.22, 0.3,0.19, 0.19, 0.7,0.2 [9,13,16,18,19,21,22] | 0.30±0.18 | 0.8, 0.5, 0.5,0.9 [18,20,21,22] | 0.68±0.21 | 0.48, 0.59 [18,27] |
|  | Bagasse | 50.0 [9] | 0.22, 0.29, 0.61, 0.22, 0.29, 0.31,0.38,1.16 [9,13,15,16,18,19,20,21] | 0.44±0.32 | 1, 1, 0.5, 0.35, 0.3,0.7 [16,18,19,20,21,22] | 0.64±0.31 | 0.48, 0.59 [18,27] |
| Sweet potato | Peelings | 77.0 [10] | 0.4, 0.35,0.5, [16,19] | 0.42±0.076 | 0.8, 0.3,0.8 [16,19,22] | 0.63±0.29 | 0.48, 0.59 [18,27] |
| Wheat | Straws | 15.0 [9] | 1.55,1.75,1.25, 1.55, 1.75, 1.80, 1.3 [9,15,16,18,19,20,22] | 1.57±0.22 | 0.53, 0.33, 0.3,0.5 [16,18,19,20,22] | 0.42± 0.12 | 0.48, 0.59 [18,27] |
|  | Husks | 15.0 [9] | 0.23,0.3 [19,22] | 0.27±0.05 | 0.29,0.3 [19,22] | 0.30±0.01 | 0.48, 0.59 [18,27] |

**Section S1.2. The eighteen treatment formulations used in estimating economically viable crop residues in Kenya**

**Table S2:** The 18 treatment formulations used in estimating economically viable crop residues, where RPR_1_ is the mean of the residue to product ratio of the i^th^ residue, RPR_2_ is the low estimate of RPR (mean minus one standard deviation) of the i^th^ residue, RPR_3_ is the high estimate of RPR (mean plus one standard deviation) of i^th^ residue. SAF_1_ is the mean of the reported values of surplus available factor for the i^th^ residue, SAF_2_ is the low estimate of SAF (mean minus one standard deviation) and SAF _3_ is the high estimate of SAF. EVF_1_ is the economically available factor and takes a value of 48% and EVF_2_ takes a value of 59%.”

|  | **Treatment formulations** |  | **Treatment formulations** |
| --- | --- | --- | --- |
| 1 | [Q, RPR_1_, SAF_1_, EVF_1_] | 10 | [Q, RPR_2_, SAF_2_, EVF_2_] |
| 2 | [Q, RPR_1_, SAF_1_, EVF_2_] | 11 | [Q, RPR_2_, SAF_3_, EVF_1_] |
| 3 | [Q, RPR_1_, SAF_2_, EVF_1_] | 12 | [Q, RPR_2_, SAF_3_, EVF_2_] |
| 4 | [Q, RPR_1_, SAF_2_, EVF_2_] | 13 | [Q, RPR_3_, SAF_1_, EVF_1_] |
| 5 | [Q, RPR_1_, SAF_3_, EVF_1_] | 14 | [Q, RPR_3_, SAF_1_, EVF_2_] |
| 6 | [Q, RPR_1_, SAF_3_, EVF_2_] | 15 | [Q, RPR_3_, SAF_2_, EVF_1_] |
| 7 | [Q, RPR_2_, SAF_1_, EVF_1_] | 16 | [Q, RPR_3_, SAF_2_, EVF_2_] |
| 8 | [Q, RPR_2_, SAF_1_, EVF_2_] | 17 | [Q, RPR_3_, SAF_3_, EVF_1_] |
| 9 | [Q, RPR_2_, SAF_2_, EVF_1_] | 18 | [Q, RPR_3_, SAF_3_, EVF_2_] |

**Section S1.3. Residue conversion factors for forest residues**

**Table S3: The ratio factors for estimating forest residues for biochar production from total wood productivity.**

This table provides information on the published values of residue generation ratio (RGR), surplus available factor (SAF) and economically available residue factor for forest residues from literature published in developing countries with specific focus in Sub-Saharan Africa.

| **Activity/ product** | **Type of residues** | **RGR** | **SAF** | **Economic available residues (t yr^-1^)** |
| --- | --- | --- | --- | --- |
| Logging | Solid wood | 0.40 [22] | 0.25 [19] | 0.48, 0.59 [18,27] |
|  | Dust | 0.20 [22] | 0.25 [19] | 0.48, 0.59 [18,27] |
| Processing | Solid wood | 0.38 [22] | 0.25 [19] | 0.48, 0.59 [18,27] |
|  | Sawdust and wood shavings | 0.28 [28] | 0.25 [19] | 0.48, 0.59 [18,27] |

**Section S1.4. The two treatment formulations used to estimate economically viable forest residues**

**Table S4:** The two treatment formulations used in estimating economically viable forest residues.

Where Q is the quantity of wood harvested or processed, RGR and SAF are the residue to product ratio and surplus available factor of respective forest residues reported in literature. EVF_1_ is the economically available factor and takes a value of 48%, while for EVF_2_ it takes a value of 59%.

|  | **Treatment formulations** |
| --- | --- |
| 1 | [Q, RGR, SAF, EVF_1_] |
| 2 | [Q, RGR, SAF, EVF_2_] |

**Section S1.5: the QGIS and R codes**

**The QGIS code for configuring marker size**

CASE

when "uncertainty_class" = 'high' then 6

when " uncertainty_class " = 'medium' then 4

ELSE 2

END

**# Perform the Wilcoxon signed-rank test crops**

test_result <- wilcox.test(data1$Total_prod_2021, data1$Total_prod_2022, paired = TRUE)

**# Print the full results**

print(test_result)

**# Perform the Wilcoxon signed-rank test forest**

test_result <- wilcox.test(data4$Year_1, data4$ Year_2, paired = TRUE)

**# Print the full results**

print(test_result)

**The R code for the Moris sensitivity analysis method**

library(sensitivity)

#Define the model function

product_model_function <- function(x) {

# The 'x' matrix has 4 columns for your 4 factors

crop_prod <- x[, 1] # crop production (tons)

RPRs <- x[, 2] # residue to product ratio

SAFs <- x[, 3] # surplus available factor

RF <- x[, 4] # economic visibility factor

# Calculate the output with a small error term

r_amount <- crop_prod * RPRs * SAFs * RF + rnorm(nrow(x), mean = 0, sd = 0.5)

return(r_amount)

}

# Define the number of factors

k <- 4

# Define the number of trajectories

r_value <- 10

# Define the minimum and maximum ranges for your factors as vectors

binf_values <- c(0.18, 0.12, 0.15, 0.48)

bsup_values <- c(2815211.44, 0.24, 1, 0.59)

# Run the Morris analysis

morris_results <- morris(model = product_model_function,

factors = k,

r = r_value,

design = list(type = "oat",

levels = 10,

binf = binf_values,

bsup = bsup_values))

print(morris_results)

plot(morris_results)

**Section S.2: Supplementary materials for results section**

**Section S.2.1: Crop and forest productivity in Kenya**

**Table S5:** Total crop productivity in all 47 counties of Kenya.

This table presents the total amount of crop production in each county in Kenya in 2021 and 2022.

| County | Productivity (1×10^5^ Mg) | |
| --- | --- | --- |
|  | 2021 | 2022 |
| Baringo | 4.67 | 4.1 |
| Bomet | 7.39 | 7.41 |
| Bungoma | 22.35 | 22.41 |
| Busia | 13.59 | 14.03 |
| Elgeyo-Marakwet | 6.28 | 5.41 |
| Embu | 4.4 | 4.38 |
| Garissa | 0.01 | 0.01 |
| Homabay | 8.13 | 8.88 |
| Isiolo | 0.21 | 0.21 |
| Kajiado | 7.59 | 8.13 |
| Kakamega | 33.99 | 25.56 |
| Kericho | 8.82 | 9.95 |
| Kiambu | 7.07 | 7.23 |
| Kilifi | 2.34 | 2.66 |
| Kirinyaga | 7.28 | 8.28 |
| Kisii | 7.19 | 7.39 |
| Kisumu | 11.75 | 12.34 |
| Kitui | 4.15 | 3.71 |
| Kwale | 4.49 | 4.42 |
| Laikipia | 1.09 | 1.33 |
| Lamu | 2.65 | 2.36 |
| Machakos | 3.69 | 3.18 |
| Makueni | 1.46 | 1.49 |
| Mandera | 0.04 | 0.04 |
| Marsabit | 0.02 | 0.03 |
| Meru | 8.89 | 8.12 |
| Migori | 11.7 | 11.55 |
| Mombasa | 0.06 | 0.06 |
| Murang’a | 8.45 | 8.94 |
| Nairobi | 0.01 | 0.02 |
| Nandi | 11.9 | 12.64 |
| Narok | 14.26 | 13.79 |
| Nyamira | 3.63 | 4.18 |
| Nyandarua | 10.21 | 11.82 |
| Nyeri | 6.46 | 6.55 |
| Samburu | 0.3 | 0.31 |
| Siaya | 2.57 | 2.78 |
| Taita-Taveta | 1.4 | 2.13 |
| Tana River | 0.49 | 0.49 |
| Tharaka-Nithi | 2.63 | 2.12 |
| Trans Nzoia | 12.54 | 11.88 |
| Turkana | 0.05 | 0.04 |
| Uasin Gishu | 11.01 | 12.36 |
| Vihiga | 7.26 | 8.97 |
| Wajir | 0.02 | 0.03 |
| West Pokot | 2.4 | 2.79 |
| Nakuru | 11.86 | 12.39 |
| **Total productivity** | **297.75** | **298.9** |

**Table S6: Total forest productivity in all 47 counties of Kenya.**

This table presents the total amount of wood production in each county in Kenya.

| Source | Type of wood | Productivity (1×10^4^ Mg) | |
| --- | --- | --- | --- |
|  |  | 2021 | 2022 |
| Locally harvested | Sawlogs and veneer logs, coniferous | 28.93 | 28.86 |
|  | Sawlogs and veneer logs, non-coniferous | 6.45 | 6.38 |
|  | Pulpwood, round and split, coniferous | 8.19 | 8.92 |
|  | Pulpwood, round and split, non-coniferous | 4.35 | 3.63 |
|  | Other industrial roundwood, coniferous | 1.34 | 2.10 |
|  | Other industrial roundwood, non-coniferous | 21.82 | 21.82 |
| Imported | Industrial roundwood, coniferous | 0.06 | 0.21 |
|  | Industrial roundwood, non-coniferous tropical | 0.21 | 0.09 |
|  | Industrial roundwood, non-coniferous non-tropical | 0.11 | 0.28 |
|  | Sawn wood, coniferous | 1.39 | 2.66 |
|  | Sawn wood, non-coniferous | 20.46 | 24.87 |
| Totals | | 93.33 | 99.80 |

**Section S.2.2: Sensitivity analysis of crop and forest residues in Kenya**

**
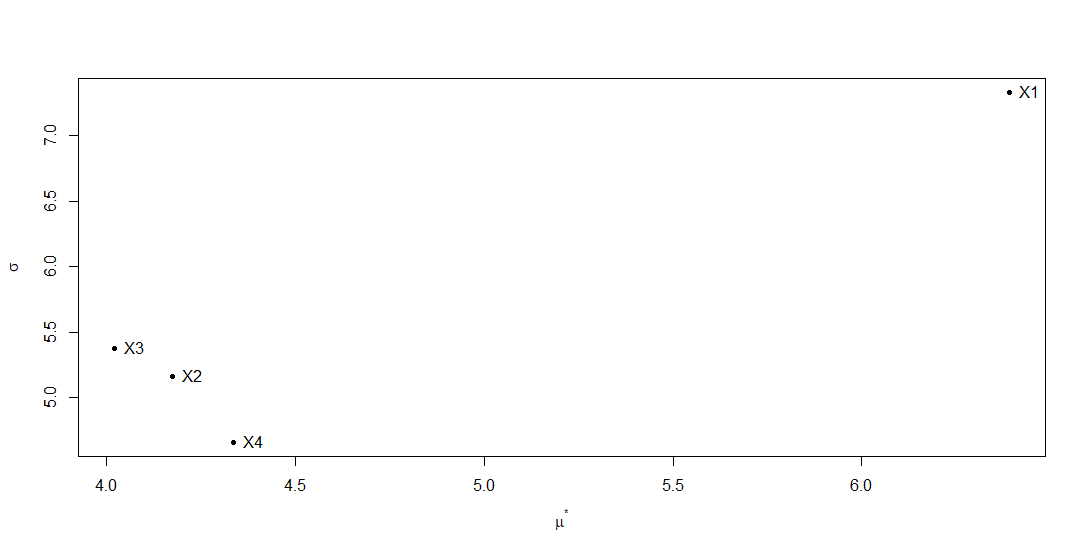
**

**Figure S1:** The Moris sensitivity analysis plot visualizing crop residue potential in Kenya, indicating absolute main effect (μ∗) against interactions (σ).

The plot indicates that crop production (X1) is the primary driver of crop residue potential in Kenya, followed by surplus available factor (X3) and residue to product ratio (X2), then economically viable factor (X4) the least influential.

**
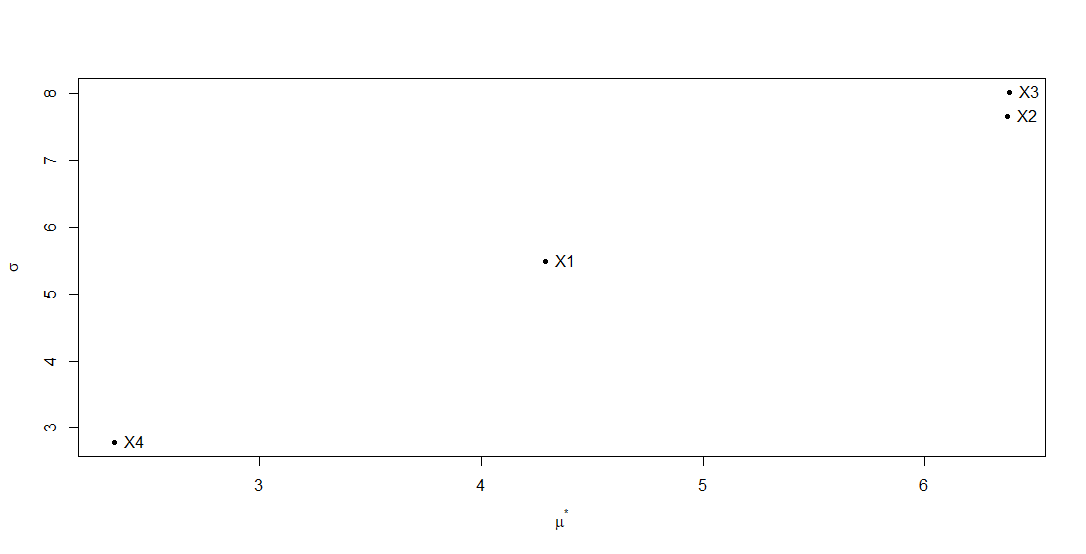
**

**Figure S2:** The Moris sensitivity analysis plot visualizing crop residue density in Kenya, indicating absolute main effect (μ∗) against interactions (σ).

The plot indicates that surplus available factor (X2) and residue to product ratio (X3) were primary drivers of crop residue densities in Kenya, followed by crop production (X1), then economically viable factor (X4) the least influential.

**
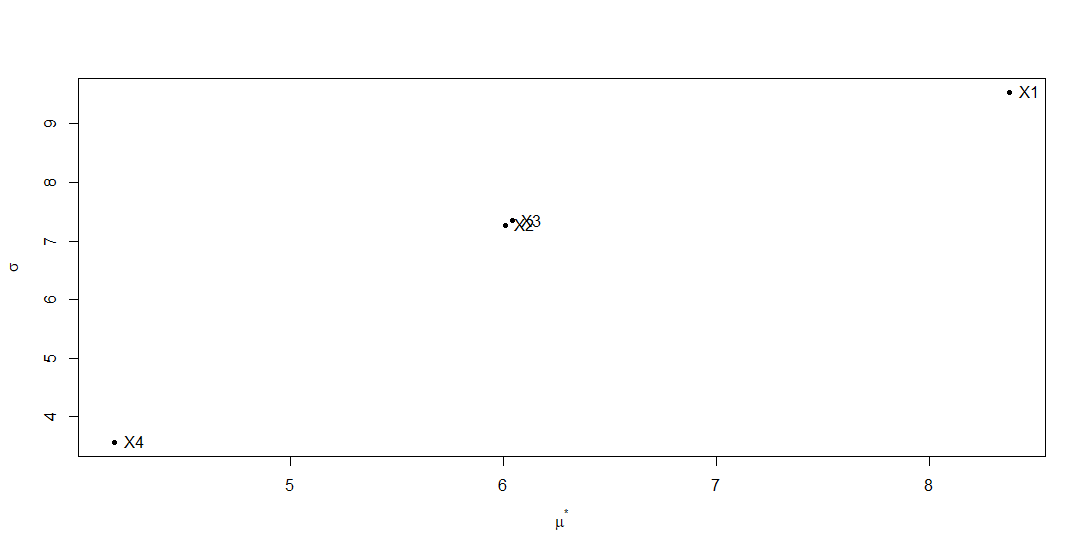
**

**Figure S3: The plot visualizing forest residue Moris sensitivity analysis of absolute main effect (μ∗) against interactions (σ) on the x-axis.** The plot indicates that forest production (X1) is the most influential factor, with surplus available factor (X3) and residue to product ratio (X2) having equal influence, then economically viable factor (X4) having the least influential).

**Section S.2.2: GIS mapping**

**Table S7: Labels and names of counties in Kenya.**

The names of counties labelled in Figure 1 and 2 in the main text are defined in this table S2.

| **County label** | **County Name** |  | **County label** | **County Name** |  | **County label** | **County name** |
| --- | --- | --- | --- | --- | --- | --- | --- |
| 1 | Mombasa |  | 17 | Makueni |  | 33 | Narok |
| 2 | Kwale |  | 18 | Nyandarua |  | 34 | Kajiado |
| 3 | Kilifi |  | 19 | Nyeri |  | 35 | Kericho |
| 4 | Tana River |  | 20 | Kirinyaga |  | 36 | Bomet |
| 5 | Lamu |  | 21 | Murang’a |  | 37 | Kakamega |
| 6 | Taita Taveta |  | 22 | Kiambu |  | 38 | Vihiga |
| 7 | Garissa |  | 23 | Turkana |  | 39 | Bungoma |
| 8 | Wajir |  | 24 | West Pokot |  | 40 | Busia |
| 9 | Mandera |  | 25 | Samburu |  | 41 | Siaya |
| 10 | Marsabit |  | 26 | Trans-Nzoia |  | 42 | Kisumu |
| 11 | Isiolo |  | 27 | Uasin Gishu |  | 43 | Homabay |
| 12 | Meru |  | 28 | Elgeyo-Marakwet |  | 44 | Migori |
| 13 | Tharaka-Nithi |  | 29 | Nandi |  | 45 | Kisii |
| 14 | Embu |  | 30 | Baringo |  | 46 | Nyamira |
| 15 | Kitui |  | 31 | Laikipia |  | 47 | Nairobi |
| 16 | Machakos |  | 32 | Nakuru |  |  |  |

**Table S8:** The amounts of crop residues in each county averaged for the two years.

This table presents the amounts of top five types of crop residues that could meet the economic criteria for biochar production in the 47 counties of Kenya. The amounts are averaged for the two years (2021 and 2022).

| **County** | **Types of crop residues** | **Economically viable (1×10^4^ Mg y^-1^)** | **Residue density (Mg y^-1^ km^-2^)** | **% contribution in the county** |
| --- | --- | --- | --- | --- |
| Baringo | Beans stalks | 4.11±1.22 | 3.74±0.24 | 11.57 |
|  | Sisal pulp | 3.57±0.10 | 3.25±0.03 | 10.05 |
|  | Maize cobs | 4.20±1.45 | 3.83±0.91 | 11.84 |
|  | Maize stalks | 17.83±2.78 | 16.24±1.62 | 50.25 |
|  | Other crop residues | 5.78±3.04 | 5.27±1.35 | 16.29 |
| Bomet | Beans stalks | 6.54±1.81 | 25.84±4.00 | 13.08 |
|  | Maize cobs | 7.25±2.37 | 28.66±3.92 | 14.51 |
|  | Maize stalks | 28.43±3.29 | 112.34±18.05 | 48.86 |
|  | Potato peels | 2.42±0.56 | 9.57±3.90 | 12.84 |
|  | Other crop residues | 5.36±2.51 | 21.16±3.03 | 10.71 |
| Bungoma | Beans stalks | 7.86±0.93 | 26.00± 2.54 | 11.83 |
|  | Maize cobs | 4.97±0.53 | 16.45±0.69 | 7.49 |
|  | Maize stalks | 19.50±2.99 | 64.47±11.83 | 29.35 |
|  | Sugarcane bagasse | 20.63±3.00 | 68.22±16.02 | 31.05 |
|  | Other crop residues | 13.48±0.98 | 44.56±12.94 | 20.28 |
| Busia | Cassava stalks | 7.34±0.58 | 43.29±1.72 | 13.17 |
|  | Rice husks | 4.34±0.84 | 25.61±0.65 | 7.79 |
|  | Maize stalks | 17.03±4.02 | 100.37±16.90 | 30.54 |
|  | Sugarcane bagasse | 10.50±3.21 | 61.91±13.03 | 18.84 |
|  | Other crop residues | 16.54±11.90 | 97.50±12.97 | 29.66 |
| Elgeyo-Marakwet | Maize cobs | 2.52±0.85 | 8.30±3.72 | 11.65 |
|  | Maize stalks | 9.86±4.01 | 32.52±12.92 | 45.65 |
|  | Potato peels | 6.08±3.21 | 20.04±5.91 | 28.14 |
|  | Beans stalks | 2.23±1.00 | 7.50±1.72 | 10.53 |
|  | Other crop residues | 0.87±0.04 | 2.87±3.60 | 4.03 |
| Embu | Banana stipples | 3.22±0.03 | 11.40±0.02 | 12.49 |
|  | Beans stalks | 2.91±0.91 | 10.30±0.23 | 14.28 |
|  | Cassava stalks | 2.89±1.93 | 10.25±6.38 | 14.23 |
|  | Maize stalks | 12.60±3.81 | 44.68±4.51 | 42.94 |
|  | Other crop residues | 4.14±6.02 | 14.66±1.92 | 16.06 |
| Garissa | Beans stalks | <0.01±0.00 | <0.01±0.00 | 9.03 |
|  | Maize cobs | <0.01±0.00 | <0.01±0.00 | 10.08 |
|  | Maize stalks | <0.01±0.00 | <0.01±0.00 | 39.53 |
|  | Sorghum straws | <0.01±0.00 | <0.01±0.00 | 28.05 |
|  | Other crop residues | <0.01±0.00 | <0.01±0.00 | 13.31 |
| Homabay | Cassava stalks | 5.25±1.85 | 16.64±11.49 | 17.46 |
|  | Maize stalks | 5.33±3.44 | 16.92±12.63 | 17.75 |
|  | Sorghum straws | 3.14±3.09 | 9.94±2.72 | 10.43 |
|  | Sugarcane bagasse | 6.58±0.84 | 20.87±22.8 | 21.89 |
|  | Other crop residues | 9.76±0.15 | 30.96±21.3 | 32.47 |
| Isiolo | Maize stalks | 0.21±0.08 | 0.08±0.06 | 27.05 |
|  | Sorghum straws | <0.01±0.01 | 0.00±0.00 | 7.88 |
|  | Wheat straws | 0.35±0.20 | 0.14±0.04 | 44.98 |
|  | Beans straws | 0.14±0.01 | 0.06±0.46 | 18.30 |
|  | Other crop residues | 0.02±0.12 | 0.01±0.00 | 1.89 |
| Kajiado | Beans stalks | 5.73±0.12 | 2.62±4.15 | 11.11 |
|  | Maize cobs | 6.35±3.27 | 2.90±3.9 | 12.32 |
|  | Maize stalks | 24.90±9.66 | 11.38±8.1 | 48.30 |
|  | Wheat straws | 5.71±0.12 | 2.61±2.09 | 11.08 |
|  | Other crop residues | 8.86±7.60 | 4.05±3.29 | 17.19 |
| Kakamega | Maiz cobs | 7.43±6.76 | 24.61±13.14 | 7.36 |
|  | Maize stalks | 29.14±10.49 | 96.48±34.36 | 28.84 |
|  | Sugarcane tops | 14.10±11.13 | 46.70±41.13 | 13.96 |
|  | Sugarcane bagasse | 37.94±11.32 | 125.62±83.67 | 37.56 |
|  | Other crop residues | 12.41±6.75 | 41.07±21.79 | 12.28 |
| Kericho | Beans stalks | 5.23±0.23 | 21.49±13.44 | 8.92 |
|  | Maize cobs | 5.85±4.34 | 24.01±24.13 | 9.96 |
|  | Maize stalks | 22.93±15.04 | 94.13±52.4 | 39.06 |
|  | Sugarcane bagasse | 12.29±3.28 | 50.43±41.52 | 20.93 |
|  | Other crop residues | 12.41±7.85 | 50.92±23.89 | 21.13 |
| Kiambu | Banana stipples | 4.473±0.28 | 17.62±4.19 | 12.18 |
|  | Coffee husks | 4.03±4.38 | 15.86±12.59 | 10.96 |
|  | Maize stalks | 17.53±12.85 | 69.07±31.17 | 47.75 |
|  | Potato peels | 3.02±1.97 | 11.90±1.96 | 8.23 |
|  | Other crop residues | 7.67±2.54 | 30.20±11.78 | 20.88 |
| Kilifi | Cassava stalks | 2.23±2.02 | 1.78±1.46 | 11.39 |
|  | Coconut husks | 1.57±0.073 | 1.25±2.09 | 8.03 |
|  | Sisal pulp | 3.71±3.15 | 2.96±2.91 | 18.94 |
|  | Maize stalks | 6.34±4.37 | 5.06±1.39 | 32.39 |
|  | Other crop residues | 5.73±1.70 | 4.57±3.98 | 29.25 |
| Kirinyaga | Potato peels | 4.68±0.43 | 31.62±22.51 | 15.99 |
|  | Coffee husks | 4.33±2.14 | 29.27±14.82 | 10.18 |
|  | Maize stalks | 18.85±9.63 | 127.50±80.81 | 36.32 |
|  | Rice husks | 3.44±0.67 | 23.29±2.92 | 14.10 |
|  | Other crop residues | 11.23±5.11 | 75.99±0.2 | 23.42 |
| Kisii | Banana stipples | 2.79±3.99 | 21.05±12.17 | 14.39 |
|  | Beans stalks | 2.85±0.75 | 21.57±4.15 | 14.65 |
|  | Maize stalks | 12.95±6.81 | 97.86±42.14 | 36.31 |
|  | Potato peels | 3.29±1.82 | 24.83±20.9 | 16.26 |
|  | Other crop residues | 4.93±2.60 | 37.25±40.02 | 18.39 |
| Kisumu | Maize stalks | 6.76±0.29 | 32.39±21.5 | 18.84 |
|  | Rice husks | 5.46±1.16 | 26.19±14.08 | 15.23 |
|  | Sorghum straws | 1.91±1.33 | 9.14±1.03 | 5.31 |
|  | Sugarcane bagasse | 14.45±6.67 | 69.28±55.28 | 40.29 |
|  | Other crop residues | 7.29±2.27 | 34.96±22.36 | 20.33 |
| Kitui | Beans straws | 2.08±2.98 | 0.69±0.72 | 11.02 |
|  | Maize cobs | 3.87±3.07 | 1.27±2.8 | 16.01 |
|  | Maize stalks | 15.59±11.46 | 5.12±5.56 | 42.46 |
|  | Sorghum straws | 3.59±0.21 | 1.18±0.41 | 15.09 |
|  | Other crop residues | 4.58±1.16 | 1.51±1.04 | 15.42 |
| Kwale | Sisal pulp | 1.66±0.01 | 2.00±3.58 | 8.53 |
|  | Maize stalks | 2.61±2.63 | 3.15±2.49 | 13.42 |
|  | Sugarcane bagasse | 1.00±1.000 | 1.18±2.61 | 21.85 |
|  | Cassava stalks | 3.70±0.19 | 4.47±1.53 | 19.02 |
|  | Other crop residues | 10.49±5.67 | 12.69±1.73 | 37.18 |
| Laikipia | Bean straws | 0.51±0.04 | 0.54±1.96 | 12.16 |
|  | Maize stalks | 2.06±1.92 | 2.16±2.19 | 37.98 |
|  | Potato peels | 0.93±1.49 | 0.97±0.75 | 19.41 |
|  | Wheat straws | 0.69±0.67 | 0.72±0.62 | 14.64 |
|  | Other crop residues | 0.85±0.67 | 0.89±1.41 | 15.81 |
| Lamu | Cassava peels | 1.29±0.21 | 2.06±0.75 | 11.69 |
|  | Cassava stalks | 3.49±1.46 | 5.58±4.04 | 27.31 |
|  | Coconut husks | 1.38±3.52 | 2.21±2.68 | 11.42 |
|  | Maize stalks | 5.57±0.60 | 8.91±6.15 | 35.01 |
|  | Other crop residues | 1.54±0.93 | 2.46±3.16 | 14.58 |
| Machakos | Beans stalks | 3.06±2.01 | 5.06±3.39 | 12.87 |
|  | Cassava stalks | 2.75±1.46 | 4.55±4.51 | 11.56 |
|  | Maize cobs | 2.96±2.45 | 4.89±2.71 | 12.44 |
|  | Maize stalks | 11.92±0.90 | 19.73±4.67 | 42.15 |
|  | Other crop residues | 3.08±1.64 | 5.10±3.24 | 20.98 |
| Makueni | Beans stalks | 1.45±3.03 | 1.77±1.11 | 6.64 |
|  | Sisal pulp | 8.61±4.16 | 10.54±4.7 | 39.50 |
|  | Sisal trunks | 2.51±0.52 | 3.07±4.66 | 11.52 |
|  | Maize stalks | 5.75±1.96 | 7.04±1.3 | 26.38 |
|  | Other crop residues | 3.48±2.67 | 4.26±3.79 | 15.97 |
| Mandera | Maize cobs | 0.04±0.55 | 0.02±0.29 | 13.69 |
|  | Maize stalks | 0.17±1.42 | 0.06±00 | 55.18 |
|  | Maize husks | 0.04±0.09 | 0.01±0.01 | 12.63 |
|  | Sorghum straws | 0.06±0.00 | 0.02±0.05 | 18.51 |
|  | Other crop residues | 0.00±0.00 | 0.00±0.00 | 0.0 |
| Marsabit | Beans straws | 0.01±0.00 | 0.00±0.00 | 6.62 |
|  | Maize cobs | 0.03±0.01 | 0.00±0.00 | 16.41 |
|  | Maize stalks | 0.13±0.26 | 0.02±0.00 | 50.16 |
|  | Sorghum straws | 0.03±0.00 | 0.00±0.00 | 15.19 |
|  | Other crop residues | 0.02±0.00 | 0.00±0.00 | 11.61 |
| Meru | Banana stipples | 11.74±5.52 | 16.75±13.6 | 38.82 |
|  | Beans stalks | 3.10±1.00 | 4.42±1.2 | 10.25 |
|  | Maize stalks | 3.80±1.85 | 5.43±1.64 | 12.57 |
|  | Potato peels | 3.48±0.45 | 4.97±2.42 | 11.50 |
|  | Other crop residues | 8.12±0.85 | 11.59±7.28 | 26.85 |
| Migori | Cassava stalks | 3.96±1.44 | 15.17±4.48 | 8.91 |
|  | Maize stalks | 15.98±8.46 | 61.15±32.37 | 35.92 |
|  | Sugarcane tops | 3.26±2.86 | 12.47±11.46 | 7.32 |
|  | Sugarcane bagasse | 8.62±1.08 | 32.99±22.35 | 19.38 |
|  | Other crop residues | 12.66±8.38 | 48.45±35.03 | 28.46 |
| Mombasa | Cassava peels | 0.05±0.00 | 2.46±3.70 | 15.25 |
|  | Cassava stalks | 0.14±0.00 | 6.56±4.91 | 40.76 |
|  | Coconut husks | 0.05±0.00 | 2.45±2.50 | 15.20 |
|  | Maize stalks | 0.04±0.00 | 1.63±1.45 | 10.11 |
|  | Other crop residues | 0.07±0.01 | 3.01±3.92 | 18.68 |
| Murang’a | Banana stipples | 3.17±1.11 | 12.54±12.03 | 24.15 |
|  | Beans stalks | 2.63±3.97 | 10.40±9.32 | 20.04 |
|  | Coffee husks | 0.94±0.53 | 3.74±3.08 | 7.21 |
|  | Maize stalks | 3.81±3.80 | 15.08±11.18 | 29.05 |
|  | Other crop residues | 2.56±3.25 | 10.15±11.53 | 19.55 |
| Nairobi | Beans stalks | 0.04±0.00 | 0.53±0.59 | 20.59 |
|  | Coffee husks | 0.02±0.02 | 0.24±0.01 | 9.43 |
|  | Maize stalks | 0.01±0.06 | 0.18±0.65 | 6.83 |
|  | Potato peels | 0.09±0.70 | 1.20±1.04 | 46.46 |
|  | Other crop residues | 0.03±0.00 | 0.04±0.06 | 16.69 |
| Nakuru | Beans stalks | 8.39±5.51 | 11.24±8.87 | 13.79 |
|  | Maize stalks | 22.76±13.92 | 30.51±14.43 | 30.43 |
|  | Potato peels | 7.49±2.11 | 10.04±12.03 | 19.32 |
|  | Wheat straws | 5.65±3.76 | 7.57±7.25 | 14.28 |
|  | Other crop residues | 16.53±12.34 | 22.15±21.12 | 22.18 |
| Nandi | Maize cobs | 5.61±3.67 | 19.65±10.19 | 10.69 |
|  | Maize stalks | 22.62±21.05 | 79.22±52.82 | 43.10 |
|  | Sugarcane tops | 5.20±3.48 | 18.20±12.32 | 9.90 |
|  | Sugarcane bagasse | 11.28±3.32 | 39.51±22.73 | 21.50 |
|  | Other crop residues | 7.77±7.07 | 27.21±24.64 | 14.80 |
| Narok | Maize stalks | 6.29±4.02 | 3.51±4.49 | 10.22 |
|  | Maize cobs | 25.38±22.08 | 14.14±4.31 | 41.19 |
|  | Sugarcane bagasse | 8.89±2.90 | 4.95±1.86 | 14.44 |
|  | Wheat straws | 5.84±11.71 | 3.25±2.75 | 9.48 |
|  | Other crop residues | 15.20±3.78 | 8.47±4.58 | 24.67 |
| Nyamira | Banana stipples | 14.64±12.70 | 163.13±91.52 | 54.36 |
|  | Beans stalks | 3.66±0.36 | 40.82±14.31 | 13.60 |
|  | Maize stalks | 3.63±3.51 | 40.46±31.12 | 13.48 |
|  | Potato peels | 3.36±4.25 | 37.45±13.39 | 12.48 |
|  | Other crop residues | 1.64±1.14 | 18.23±15.57 | 6.08 |
| Nyandarua | Beans straws | 3.22±4.58 | 9.80±3.38 | 6.61 |
|  | Maize cobs | 5.58±1.70 | 16.99±11.67 | 11.46 |
|  | Maize stalks | 18.61±11.84 | 56.65±44.48 | 38.22 |
|  | Potato peels | 8.72±2.23 | 26.53±20.44 | 17.90 |
|  | Other crop residues | 12.57±6.28 | 38.25±12.83 | 25.80 |
| Nyeri | Banana stipples | 5.71±0.07 | 17.17±13.07 | 15.26 |
|  | Beans stalks | 4.32±2.51 | 12.99±2.49 | 22.31 |
|  | Maize stalks | 14.59±11.28 | 43.87±33.46 | 39.00 |
|  | Potato peels | 3.67±4.32 | 11.02±0.41 | 16.12 |
|  | Other crop residues | 9.13±5.20 | 27.44±31.92 | 7.31 |
| Samburu | Beans stalks | 0.33±0.15 | 0.16±0.79 | 17.54 |
|  | Maize cobs | 0.21±0.21 | 0.10±0.03 | 11.23 |
|  | Maize stalks | 0.84±1.51 | 0.40±3.66 | 45.26 |
|  | Wheat straws | 0.24±1.44 | 0.11±0.91 | 12.88 |
|  | Other crop residues | 0.24±0.95 | 0.12±2.1 | 13.10 |
| Siaya | Beans stalks | 2.14±0.76 | 8.44±2.52 | 11.88 |
|  | Maize cobs | 2.30±1.69 | 9.11±8.44 | 12.82 |
|  | Maize stalks | 5.82±4.95 | 22.99±13.29 | 32.09 |
|  | Sugarcane bagasse | 4.15±2.89 | 16.40±3.84 | 23.08 |
|  | Other crop residues | 3.57±3.93 | 14.11±7.45 | 20.13 |
| Taita Taveta | Banana stipples | 1.66±1.01 | 0.97±3.93 | 8.48 |
|  | Sisal pulp | 11.96±12.81 | 6.97±3.78 | 61.01 |
|  | Sisal trunk | 3.49±1.34 | 2.03±1.85 | 17.80 |
|  | Maize stalks | 0.88±2.52 | 0.52±0.15 | 4.51 |
|  | Other crop residues | 1.61±1.67 | 0.94±0.46 | 8.20 |
| Tana River | Beans straws | 0.61±0.20 | 0.16±0.84 | 18.83 |
|  | Maize cobs | 0.21±0.87 | 0.06±0.06 | 6.61 |
|  | Maize stalks | 0.86±3.50 | 0.23±0.04 | 26.64 |
|  | Rice husks | 1.05±1.50 | 0.28±1.43 | 32.63 |
|  | Other crop residues | 0.49±2.93 | 0.13±1.64 | 15.30 |
| Tharaka-Nithi | Banana stipples | 0.75±3.16 | 2.94±3.83 | 9.18 |
|  | Cassava stalks | 1.91±2.12 | 7.46±3.91 | 23.29 |
|  | Maize stalks | 2.33±0.37 | 9.08±2.63 | 28.36 |
|  | Sorghum straws | 1.14±4.08 | 4.44±2.06 | 13.88 |
|  | Other crop residues | 2.08±0.01 | 8.10±4.47 | 25.30 |
| Trans Nzoia | Beans stalks | 5.94±2.93 | 23.80±23.29 | 7.10 |
|  | Maize cobs | 11.86±7.33 | 47.53±31.38 | 14.18 |
|  | Maize stalks | 47.82±25.53 | 191.64±103.48 | 57.17 |
|  | Maize husks | 11.00±12.75 | 44.08±17.22 | 13.15 |
|  | Other crop residues | 7.02±1.69 | 28.14±22.02 | 8.39 |
| Turkana | Beans straws | 0.03±0.18 | 0.00±0.00 | 6.86 |
|  | Maize cobs | 0.03±0.46 | 0.00±0.00 | 7.46 |
|  | Maize stalks | 0.11±0.00 | 0.02±0.08 | 30.07 |
|  | Sorghum straws | 0.21±0.14 | 0.03±0.59 | 55.61 |
|  | Other crop residues | 0.00±0.00 | 0.00±0.00 | 0.00 |
| Uasin Gishu | Maize cobs | 11.60±4.09 | 34.17±10.64 | 14.36 |
|  | Maize stalks | 46.74±42.80 | 137.79±92.38 | 57.88 |
|  | Maize husks | 10.74±4.65 | 31.65±11.69 | 13.30 |
|  | Potato peels | 3.14±4.40 | 9.26±8.86 | 3.89 |
|  | Other crop residues | 8.54±0.65 | 25.18±22.7 | 10.58 |
| Vihiga | Beans stalks | 5.47±0.51 | 0.01±0.01 | 7.99 |
|  | Maize cobs | 10.41±2.27 | 184.68±101.69 | 15.21 |
|  | Maize stalks | 41.98±31.021 | 744.60±203.06 | 61.34 |
|  | Cassava stalks | 9.63±1.33 | 170.77±132.16 | 14.07 |
|  | Other crop residues | 0.95±0.85 | 16.92±4.24 | 1.39 |
| Wajir | Maize cobs | 0.40±0.83 | 0.01±0.07 | 15.85 |
|  | Maize stalks | 0.16±1.40 | 0.03±0.04 | 63.92 |
|  | Maize husks | 0.04±0.08 | 0.01±0.03 | 14.63 |
|  | Sorghum straws | 0.01±0.07 | 0.00±0.00 | 5.58 |
|  | Other crop residues | <0.01±0.00 | 0.00±0.00 | 0.02 |
| West Pokot | Cassava stalks | 1.45±2.35 | 1.59±1.80 | 9.64 |
|  | Maize cobs | 1.98±3.03 | 2.17±2.72 | 13.18 |
|  | Maize stalks | 7.98±3.11 | 8.75±2.02 | 53.13 |
|  | Sorghum straws | 1.83±1.88 | 2.01±3.51 | 12.20 |
|  | Other crop residues | 1.78±1.45 | 1.95±1.82 | 11.86 |

**References**

[1] Khan, M.K., & Perveen, B. Transformation of agricultural wastes into sugar by Trichoderma viride. *J. Pure Appl. Microbiol*. **4**, 103-108 (2010).

[2] Lawrence, M., Heath, A., & Walker, P. *Monitoring of the moisture content of straw bale walls*. In: Howlett, R.J., Jain, L.C., Lee, S.H. (Eds.) Sustainability in Energy and Buildings. (Springer, Berlin, Heidelberg 2009).

[3] Nwoko, C. I., Enyinnaya, O. C., Okolie, J. I., & Nkwoada, A. The proximate analysis and biochemical composition of the waste peels of three cassava cultivars. *Int. J. Sci. Eng. Appl. Sci.* ***2***, 64–71. (2016).

[4] Kaewwinud, N., Khokhajaikiat, P., & Boonma, AEffect of moisture and region of cut on cassava stalk properties in biomass applications. *Res. Agr. Eng.* **63**, 23–28; [https://doi.org/10.17221/70/2015-RAE](https://www.google.com/search?q=https://doi.org/10.17221/70/2015-RAE) (2017).

[5] Ighalo, J. O. et al. Biochar from coconut residues: An overview of production, properties, and applications. *Ind. Crops Prod.* **204**, 117300; <https://doi.org/10.1016/j.indcrop.2023.117300> (2023).

[6] Obeng, G.Y. et al. Coconut wastes as bioresource for sustainable energy: Quantifying wastes, calorific values and emissions in Ghana. *Energies*. **13**, 2178; https://doi.org/10.3390/en13092178 (2020).

[7] Jaramillo, H.Y., Vasco-Echeverri, O., & Camperos, J.A.G. Characterization of the coffee husk: a potential alternative for sustainable construction. *Civ. Eng. Archit.* **11**, 1902–1908; <https://doi.org/10.13189/cea.2023.110418> (2023).

[8] Maduako, J. N., & Hamman, M. Determination of some physical properties of three groundnut varieties. *Niger. J. Technol.* **24**, 12–17 (2004).

[9] Kimutai, A. Muumbo, A., Siagi, Z. & Kiprop, A. A study on agricultural residues as a substitute to fire Wood in Kenya: a review on major crop. *J. Energy Technol. Policy.* **4**, 45-51 (2014).

[10] Kinoka, M. Potato and potato peels: A comprehensive review on potato peels nutritional composition, bioactive compounds and its application in the food industry. *J. Pharm. Innov.* **12**, 182–187 (2023).

[11] Muthangya, M., Hashim, S.O., Amana, J.M., Mshandete, A.M., & Kivaisi, A.K. Auditing and characterisation of sisal processing waste: A bioresource for value addition. *Afr. J. Account. Soc. Sci. Stud.* ***2***, 52–61 (2019).

[12] Tanyi, R.J. & Adaramola, M.S. Bioenergy potential of agricultural crop residues and municipal solid waste in Cameroon. *AIMS Energy*. **11**, 31–46; <https://doi.org/10.3934/energy.2023002> (2023).

[13] Suryani, A., Bezama, A., Mair-Bauernfeind, C., Makenzi, M. & Thrän, D. Drivers and barriers to substituting firewood with biomass briquettes in the Kenyan tea industry. *Sustainability.* **14**, 5611; <https://doi.org/10.3390/su14095611> (2022).

[14] Zanli, L.B., Gbossou, K.C., Tang, W., Kamoto, M. & Chen, J. A review of biochar potential in Cote d’Ivoire in light of the challenges facing Sub-Saharan Africa. *Biomass Bioenergy.* **165**, 10658; <https://doi.org/10.1016/j.biombioe.2022.106581> (2022).

[15] Jekayinfa, S.O, Orisaleye, J.I. & Pecenka, R. An assessment of potential resources for biomass energy in Nigeria. *Resources,* **9**, 92, <https://doi.org/10.3390/resources9080092> (2020).

[16] Ukoba, M.O. et al. Geographic information systems (GIS) approach for assessing the biomass energy potential and identification of appropriate biomass conversion technologies in Nigeria. *Biomass and Bioenergy*. **170**, 106726; <https://doi.org/10.1016/j.biombioe.2023.106726> (2023).

[17] Ortiz-Ulloa, J.A., Abril-González, M.F., Pelaez-Samaniego, M.R., & Zalamea-Piedra, T.S. Biomass yield and carbon abatement potential of banana crops (*Musa spp*.) in Ecuador. *Environ. Sci. Pollut. Res. Int.* **28**, 18741–18753; <https://doi.org/10.1007/s11356-020-09755-4> (2021).

[18] Souza, L.P. et al. Theoretical and technical assessment of agroforestry residue potential for electricity generation in Brazil towards 2050. *Energy Rep*. **7**, 2574–2587; <https://doi.org/10.1016/j.egyr.2021.04.026> (2021).

[19] Gabisa, E.W. & Gheewala, S.H. Potential of bio-energy production in Ethiopia based on available biomass residues. *Biomass and Bioenergy.* **111,** 77-87; <https://doi.org/10.1016/j.biombioe.2018.02.009> (2018).

[20] Portugal-Pereira, J., Soria, R., Rathmann, R., Schaeffer, R. & Szklo, A. Agricultural and agro-industrial residues-to-energy: Techno-economic and environmental assessment in Brazil. *Biomass and Bioenergy*. **81**, 521-533; <https://doi.org/10.1016/j.biombioe.2015.08.010> (2015).

[21] Rhofita, E.I., Rachmat, R., Meyer, M. & Montastruc, L. Mapping analysis of biomass residue valorization as the future green energy generation in Indonesia. *J. Clean. Prod.* **354**, 131667; <https://doi.org/10.1016/j.jclepro.2022.131667> (2022).

[22] Tolessa, A. Bioenergy production potential of available biomass residue resources in Ethiopia. *Renew. Energy.* **2023**, 2407300; <https://doi.org/10.1155/2023/2407300> (2023).

[23] Dasappa, S. Potential of biomass energy for electricity generation in sub-Saharan Africa. *Energy Sustain. Dev.* **15,** 203-213; <https://doi.org/10.1016/j.esd.2011.07.006> (2011).

[24] Terrapon-Pfaff, J.C., Fischedick, M. & Monheim, H. Energy potentials and sustainability—the case of sisal residues in Tanzania. *Energy Sustain. Dev*, **16,** 312-319; <https://doi.org/10.1016/j.esd.2012.06.001> (2012).

[25] NIRAS-LTS., E4tech., AIGUASOL. & Aston University. *Bioenergy for sustainable local energy services and energy access in Africa, demand sector report 7: Sisal processing, Kenya*. (For Carbon Trust and UK Government, 2021).

[26] Pippo, W., Luengo, C., Lidice, A., Gilberto, G.P., & Neto, J. Energy recovery from sugarcane: Study of heating value variations of sugarcane-trash with moisture content during the milling season. *Waste Biomass Valor.* **2**, 1–16; <https://doi.org/10.7726/ajbb.2014.1001> (2011).

[27] Haase, M., Rösch, C., & Ketzer, D. GIS-based assessment of sustainable crop residue potentials in European regions. *Biomass Bioenergy*. **86**, 156-171; <https://doi.org/10.1016/j.biombioe.2016.01.020> (2016).

[28] Mydlarz, K.; Wieruszewski, M. Economic, technological as well as environmental and social aspects of local use of wood by-products generated in sawmills for energy purposes. *Energies*. **15**, 1337; <https://doi.org/10.3390/en15041337> (2022).
